# Transcript Profiling Identifies Gene Cohorts Controlled by Each Signal Regulating *Trans*-Differentiation of Epidermal Cells of *Vicia faba* Cotyledons to a Transfer Cell Phenotype

**DOI:** 10.3389/fpls.2017.02021

**Published:** 2017-11-28

**Authors:** Hui-Ming Zhang, Simon L. Wheeler, Xue Xia, Kim Colyvas, Christina E. Offler, John W. Patrick

**Affiliations:** ^1^School of Environmental and Life Sciences, University of Newcastle, Callaghan, NSW, Australia; ^2^School of Mathematical and Physical Sciences, University of Newcastle, Callaghan, NSW, Australia

**Keywords:** RNA-sequencing, signaling, cell wall, transfer cells, wall ingrowth papillae, wall labyrinth

## Abstract

Transfer cells (TCs) support high rates of membrane transport of nutrients conferred by a plasma membrane area amplified by lining a wall labyrinth comprised of an uniform wall layer (UWL) upon which intricate wall ingrowth (WI) papillae are deposited. A signal cascade of auxin, ethylene, extracellular hydrogen peroxide (H_2_O_2_) and cytosolic Ca^2+^ regulates wall labyrinth assembly. To identify gene cohorts regulated by each signal, a RNA- sequencing study was undertaken using *Vicia faba* cotyledons. When cotyledons are placed in culture, their adaxial epidermal cells spontaneously undergo *trans*-differentiation to epidermal TCs (ETCs). Expressed genes encoding proteins central to wall labyrinth formation (signaling, intracellular organization, cell wall) and TC function of nutrient transport were assembled. Transcriptional profiles identified 9,742 annotated ETC-specific differentially expressed genes (DEGs; Log_2_fold change > 1; FDR p ≤ 0.05) of which 1,371 belonged to signaling (50%), intracellular organization (27%), cell wall (15%) and nutrient transporters (9%) functional categories. Expression levels of 941 ETC-specific DEGs were found to be sensitive to the known signals regulating ETC *trans*-differentiation. Significantly, signals acting alone, or in various combinations, impacted similar numbers of ETC-specific DEGs across the four functional gene categories. Amongst the signals acting alone, H_2_O_2_ exerted most influence affecting expression levels of 56% of the ETC-specific DEGs followed by Ca^2+^ (21%), auxin (18%) and ethylene (5%). The dominance by H_2_O_2_ was evident across all functional categories, but became more attenuated once *trans*-differentiation transitioned into WI papillae formation. Amongst the eleven signal combinations, H_2_O_2_/Ca^2+^ elicited the greatest impact across all functional categories accounting for 20% of the ETC-specific DEG cohort. The relative influence of the other signals acting alone, or in various combinations, varied across the four functional categories and two phases of wall labyrinth construction. These transcriptome data provide a powerful information platform from which to examine signal transduction pathways and how these regulate expression of genes encoding proteins engaged in intracellular organization, cell wall construction and nutrient transport.

## Introduction

Transfer cells (TCs) differentiate at cellular sites supporting high rates of apo-/symplasmic exchange of nutrients. These sites can include interfaces between soil/root, maternal/filial tissues of developing seeds and biotroph/plant host as well as loading/unloading regions of phloem and xylem (Offler et al., 2003). The TC capacity for apo-/symplasmic exchange of nutrients is conferred by development of a wall labyrinth that forms a scaffold supporting an amplified (10- to 20-fold) plasma membrane area enriched in transporters. As a result, TCs can contribute to plant productivity and crop yield by enhancing overall (e.g., soil/root interface – Schmidt and Bartels, 1996) or directing preferential (e.g., developing seeds – Andriunas et al., 2013) nutrient flows to nourish whole plant or selective organ development respectively. Equally, TCs can compromise plant productivity and crop yield in those circumstances where the invading pathogen (e.g., root knot nematodes – Cabrera et al., 2014) has hijacked the developmental program responsible for constructing the TC phenotype to support their nutritional requirements. Thus, in the quest to improve crop yield and crop protection, there are compelling imperatives to discover the cell and molecular mechanisms underpinning TC development.

A major impediment to discovering cell and molecular mechanisms underpinning TC development is that, in most circumstances, TCs are embedded within tissue matrices and occur in low numbers (Offler et al., 2003). An experimental system that overcomes this technical impasse is one in which 90% of the adaxial epidermal cell population of developing *Vicia faba* cotyledons spontaneously undergo quasi-synchronous *trans*-differentiation to a TC phenotype upon excision and transfer to a culture medium (Offler et al., 1997; Wardini et al., 2007). The phenotype of the adaxial epidermal TC (ETC), structurally and functionally mirrors that of their abaxial ETC counterparts (Farley et al., 2000; Talbot et al., 2001). Their location on the cotyledon surface in large numbers (thousands) and ready isolation in epidermal peels has opened up opportunities for replicated cell (both live and preserved) and molecular biology investigations of their development (Andriunas et al., 2013). A complex of sequential signals comprising auxin (Dibley et al., 2009), ethylene (Zhou et al., 2010) negatively regulated by glucose through a hexokinase pathway (Andriunas et al., 2011), extracellular H_2_O_2_ (Andriunas et al., 2012; Xia et al., 2012) and cytosolic Ca^2+^ (Ca^2+^; Zhang et al., 2015a) participate in regulating construction of the wall labyrinth comprised of an UWL upon which WI papillae form (Andriunas et al., 2013). The phasic temporal development of the UWL followed by that of WI papillae has permitted identification of the ETC-specific transcriptomes associated with each of these phases of wall labyrinth construction (Zhang et al., 2015d; Arun-Chinnappa and McCurdy, 2016). Comparable temporal studies have been undertaken on developing basal endosperm TCs of barley (Thiel, 2014) and maize (Zhan et al., 2015) grains as well as on nematode-induced giant cells formed in roots (Cabrera et al., 2014, 2016). Collectively this work has identified TC-specific genes encoding signal cascades (Yuan et al., 2016) and downstream machinery responsible for constructing the TC wall labyrinth and membrane transporters that confer TC function (Cabrera et al., 2014; Thiel, 2014; Zhan et al., 2015; Zhang et al., 2015d). A significant outstanding question is to discover the cohorts of downstream genes regulated by each of the various signaling cascades.

As a first step in filling this knowledge gap described above, we designed a RNA-seq experiment to identify gene cohorts controlled by each signal currently known to participate in regulating ETC *trans*-differentiation (i.e., auxin, ethylene, H_2_O_2_ and Ca^2+^) of cultured *V. faba* cotyledons. To this end, cotyledons were cultured on media in the absence (control) and presence (treated) of pharmacological blockers of each signal. Transcriptome comparisons between ETC and underlying SPC, obtained from control cotyledons, identified the assemblage of DEGs specifically expressed in the *trans*-differentiating ETCs. The analysis was further refined by identifying those ETC-specific DEGs that were located in functional categories (Mapman and KEGG) considered to contribute to wall labyrinth construction (i.e., signaling, intracellular organization and cell wall) and to TC function in nutrient transport. Numbers of ETC-specific DEGs in these TC functional categories regulated by one or more signals were deduced from determining whether their transcript levels were significantly altered in cotyledons cultured on each of the pharmacological blockers. Overall, the findings demonstrated that signals acting alone exerted control over the greater number of ETC-specific DEGS during UWL construction while various signal combinations assumed dominance during the subsequent developmental phase of WI papillae formation. H_2_O_2_, acting alone or in combination with other signals, regulated expression of the greatest number of ETC-specific DEGs during both phases of wall labyrinth construction and within each of the four gene functional categories examined. Numbers of ETC-specific DEGs influenced by the remaining signals in descending order, were Ca^2+^, auxin and ethylene with H_2_O_2_/Ca^2+^ being the strongest signal combination.

## Materials and Methods

### Plant Growth Conditions, Cotyledon Culture and Collection of Tissue Samples for Sequencing

*Vicia faba* L. (cv. Fiord) plants were raised under controlled environmental conditions (Dibley et al., 2009). Cotyledons were freshly harvested or cultured for specified times on liquid MS medium containing ± specified pharmacological agents to block signal action (see Experimental Design for choice of culture time and agents). Each pharmacological agent was applied at a concentration that did not impact cell viability as verified by staining tissue sections of cultured cotyledons with 0.1% (w/v) tetrazolium blue for 20 min. At harvest, cotyledons were immediately fixed in 75% ethanol and 25% acetic acid for 1 h at 4 °C. Peels of the *trans*-differentiating adaxial ETCs were collected from replicate batches of fixed cotyledons exposed to each pharmacological treatment and pooled (ca 16 mg fresh weight of epidermal peels/replicate batch), immediately snap-frozen in liquid nitrogen and stored at -80 °C until used for RNA extraction (Dibley et al., 2009).

### RNA Isolation, cDNA-Library Construction and Illumina Sequencing

Total RNA was extracted using Qiagen (USA) RNeasy plant mini kits. Contaminating genomic DNA was removed using DNase I. Total RNA quality was verified by determining the integrity of the 25S and 18S RNA with an Agilent 2100 Bioanalyzer (Agilent, United States; Zhang et al., 2015d).

cDNA libraries were prepared from poly-A mRNA isolated from 1 μg of total RNA of each biological replicate using a TruSeq^®^ RNA V2 sample preparation kit (Illumina, United States) according to the manufacturer’s instructions. cDNA quality was evaluated by determining size and purity using an Agilent 2100 Bioanalyzer. cDNA fragments, ranging from 100 to 700 bp, were selected by agarose gel purification and indexed with unique nucleic acid identifiers (Illumina TruSeq V2 index sequence). The indexed cDNA libraries were diluted to an average concentration of 10 nM and pooled in equal volumes (10 μL of each library) to generate the final mixed cDNA pool for sequencing. The pool was then sequenced using 100 bp pair-end sequencing strategy on an Illumina HiSeq 2000 platform (Australian Genome Research Facility, Melbourne, VIC, Australia).

### Filtering, Mapping and Annotation of Sequenced Reads

Using Illumina CASAVA pipeline version 1.8.2, raw reads were trimmed with adaptor filtering and a read length cut-off of 50%. Thereafter, filtered reads with over 20% of their nucleotides having a *Q* score < 20 (probability of sequencing error > 0.01) or their sequences having an N reading over 5% were removed. Filtered reads were mapped to the previously constructed transcriptome library of *trans*-differentiating adaxial ETCs of *V. faba* cotyledons (Zhang et al., 2015d) using SOAPallgner/SOAP2 (BGI, China) to obtain abundance of transcripts, normalized as RPKM.

All mapped unigenes were re-annotated using the Mercator pipeline^1^ (Lohse et al., 2014) against the following publically available databases: The Arabidopsis Information Resource (TAIR10) proteins (Swarbreck et al., 2010), SwissProt/Uniprot plant proteins^2^, CDD (Marchler-Bauer et al., 2007), COG^3^, JGI Chlamy release 4 Augustus models (CHLAMY; Nordberg et al., 2014), TIGR5 rice proteins (ORYZA; Kawahara et al., 2013) and InterProScan (Zdobnov and Apweiler, 2001). A KEGG^4^ enzymatic pathway annotation was also performed. In all annotations, an *e*-value threshold of 1e^-5^ was used as a cut-off for meaningful annotation.

### Differentially Expressed Gene Analysis and Other Statistical Tests

Any outlier replicate RPKM values within each culture time/treatment were identified (Grubbs, 1969) and removed. Only genes exhibiting mean RPKM values > 1 were retained for further analysis. DEGs between culture times of control cotyledons were identified in the *trans*-differentiating ETCs using limmaR (Ritchie et al., 2015) and in SPCs employing edgeR (McCarthy et al., 2012; rationale outlined in the Experimental Design section). Genes were considered differentially expressed if their Log_2_-fold change was greater than 1 with a FDR of *p* ≤ 0.05 (Benjamini and Hochberg, 1995).

Impacts of blocking inductive signals on TC-specific DEGs were assessed by comparing RPKM values for control against those for each signal using lmFit function in limmaR to conduct independent sample t-tests, with a two-tailed probability value threshold of 0.05 and no FDR restriction.

### Quantitative RT-PCR Validation

Aliquots from the RNA samples sent for Illumina sequencing were converted to cDNA using the QuantiTect Reverse Transcription Kit (Qiagen, United States). Primers were designed using Primer 3 plus (Whitehead Institute for Biomedical Research, United States) and synthesized by Sigma–Aldrich Australia (see Supplementary Table S1 for primer sequences). For each qRT-PCR reaction, a 15 μL mix containing 7.5 μL SYBR Green master mix (Qiagen, United States), 0.375 μL of forward and reverse primers (10 μM), 1.75 μL of nuclease free H_2_O and 5 μL cDNA was set up. The following PCR cycle was used: 95°C for 5 min, 95°C for 15 s, 60°C for 20 s, 72°C for 30 s; steps 2–4 were repeated 50 times. High-resolution melting curves (72–95°C) following the final PCR cycle checked the specificity of the PCR products. For each cDNA sample, technical duplicates for each biological replicate (*n* = 3) were tested.

Four housekeeping gene candidates, *elongation factor 2-α* (*VfEF2α)*, *NADH dehydrogenase subunit 4* (*VfNADHD4*), *60S ribosomal protein subunit L2* (*Vf60SL2*), and *multi-domain cyclophilin type peptidyl-prolyl cis-trans isomerase G* (*VfPPlaseG*) were selected based on their stable expression in adaxial epidermal cells during TC *trans*-differentiation (Supplementary Figure S1). These candidates were further assessed (see Supplementary Table S1 for primer sequences) using GeNorm analysis of real-time PCR data derived from cDNA of epidermal peels that collectively yielded a reliable reference *M* value of <0.15 (Vandesompele et al., 2002). Relative expression levels of each unigene were determined using the ΔΔct method.

### Scanning Electron Microscopy

Protoplasts of adaxial epidermal peels (Dibley et al., 2009) were removed by washing in 2% (v/v) NaClO for 3 h at room temperature with hourly changes followed by three 10-min washes in dH_2_O to remove the bleach. Peels then were placed in small steel cages and dehydrated at 4°C through a 10% step-graded ethanol/dH_2_O series, changed at 30-min intervals from 10% up to 100% ethanol. After holding in 100% ethanol at least overnight, peels were critical-point dried with liquid CO_2_ in a critical-point drier (Balzers Union, Liechtenstein). The dried peels were orientated outer face down on sticky carbon tabs to reveal the cytoplasmic face of their outer periclinal cell walls. Samples were sputter coated with gold to a thickness of 20 nm in a sputter-coating unit (SPI Suppliers, United States) and viewed at 15 kV with a Philips XL30 scanning electron microscope.

## Results and Discussion

### Experimental Design

#### Selection of Cotyledon Culture Times and Pharmacological Agents for RNA-Sequencing

**FIGURE 1:**
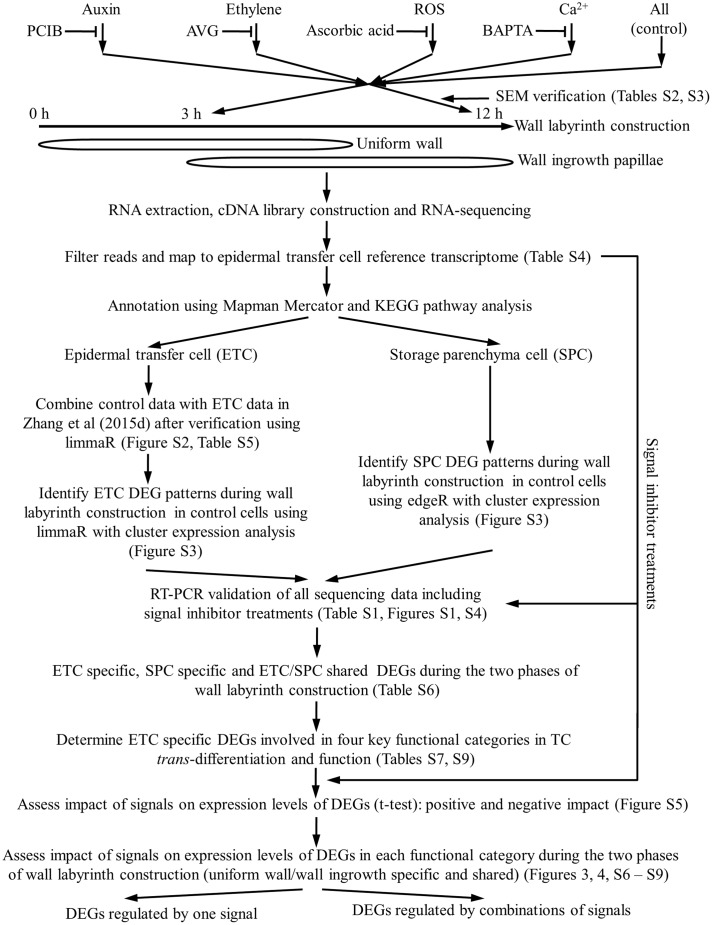
Schematic flow chart illustrating the experimental design used to investigate signal regulation of the transcriptional network underpinning TC *trans*-differentiation and function.

Cotyledons were cultured for specified times to capture transcriptional activities during the two phases of wall labyrinth construction. Freshly harvested cotyledons were used as a baseline transcriptome of the adaxial epidermal cells before their induction to undergo *trans*-differentiation to a TC-morphology (**Figure 1**). A 3-h culture time was chosen as representative of the transcriptome during UWL formation with >40% of the *trans*-differentiating ETCs exhibiting an UWL (Zhang et al., 2015d) while WI papillae formation was evident in <10% of cells (Wardini et al., 2007). Following 12 h of cotyledon culture, UWL formation has ceased (Zhang et al., 2015d) while WI papillae construction continues in over 75% of cells (Wardini et al., 2007). Thus 12-h cultured cotyledons offer a transcriptome representative of *trans*-differentiating ETCs supporting WI papillae construction alone (**Figure 1**).

The four signals (auxin, ethylene, H_2_O_2_ and Ca^2+^) identified to date as responsible for regulating *trans*-differentiation to a TC-morphology, were blocked using pharmacological agents known to be effective in the *V. faba* cotyledon culture system (**Figure 1** and, for more details, see Supplementary Table S2). Cotyledons were either freshly harvested, or cultured in the absence/presence of the signal blockers (Supplementary Table S2), for 4 h at 4°C to allow the blocker to enter the cotyledons prior to their epidermal cells being rapidly induced to undergo *trans*-differentiation following transfer to 26°C for a further 3 or 12 h of culture (see for example, Zhang et al., 2015b). To verify that each set of cultured cotyledons was *trans*-differentiating and responding to the pharmacological blockades, percentages of ETCs forming WI papillae of a sub-set of cotyledons at 12 h of culture were scored along with ETC viability (Supplementary Table S3). Only those biological replicate batches of cotyledons that complied with previously reported responses of WI papillae formation to the pharmacological agents (Supplementary Table S3) were processed for RNA-seq analysis to determine their transcriptomes (**Figure 1**). That the pharmacological agents specifically block ETC *trans*-differentiation has been demonstrated previously whereby an exogenous supply of each signal fully restored WI papillae formation in the presence of the respective pharmacological blockade (see Supplementary Table S3).

#### Preliminary Analysis of RNA-Seq Reads and Strategy to Identify TC-Specific Differentially Expressed Genes

Fifty-nine to 71 million clean reads were generated for each biological replicate (Supplementary Table S4; Zhang et al., 2015d). Unigene transcript abundance was determined for only uniquely mapped reads (58% of total mapped reads) normalized as RPKM values. All unigenes then had their biochemical functions annotated using Mapman Mercator and KEGG pathway analyses.

To increase the statistical power of the control RNA-seq RPKM data set, we evaluated whether these data could be combined with that reported in Zhang et al. (2015d). Multidimensional scaling plots of these two data sets using limmaR (Ritchie et al., 2015) showed tight coupling of gene expression profiles between the two data sets at each time point (Supplementary Figure S2). Further analysis using limmaR DEG analysis showed that between these two data sets, at each time point, >99% of genes shared identical expression levels (FDR corrected *p* > 0.05; Supplementary Table S5). For transcripts fulfilling this criterion, their RPKM values from the two data sets were combined increasing replicate numbers to six. Those not satisfying this criterion were excluded from further analysis (**Figure 1**).

Temporal changes of control RPKM values of unigenes expressed in ETCs were assessed using limmaR (for details see Materials and Methods). Nine temporal expression profile patterns were identified for up- and down-regulated DEGs (Supplementary Figure S3). These patterns were aggregated into three broad temporal expression profiles of UWL specific (0–3 h), WI papillae specific (3 – 12 h) and shared between the two phases of wall labyrinth construction. In each temporal expression profile, DEGs that were up- or down-regulated are presented separately (**Figure 1** and **Table 1**). To identify ETC-specific DEGs in these profiles, a similar analysis of temporal gene expression was undertaken for the SPCs. To ensure a conservative identification of ETC-specific DEGs, the rigor of limmaR analysis applied to the epidermal cell data set was relaxed somewhat using edgeR (McCarthy et al., 2012) to identify DEGs in the SPCs (**Figure 1** and Supplementary Figure S3). Comparison of the temporal expression profiles for the two cell types in each of the three broad temporal expression profiles, identified DEGs that were ETC-specific, SPC specific and shared between the two cell types (**Figure 1**, **Table 1**, and Supplementary Table S6).

**Table 1:** Global identification of DEGs in *trans*-differentiating ETCs expressed at the two phases of wall labyrinth construction.

Temporal expression profile coincides with	Pattern of DEGs	Total number of DEGs	ETC specific DEGs	Annotated ETC specific DEGs	Functional DEGs related to TC *trans*-differentiation	Functional DEGs regulated by known signals
UWL deposition (0–3 h specific)	Up-regulated	3809	3208	1581	412	265


	Down-regulated	3394	3296	1655	369	227
WI deposition (3–12 h specific)	Up-regulated	834	733	446	130	99


	Down-regulated	314	307	146	42	31
Shared UWL/WI (0–3 h and 3–12 h)	Up-regulated	1709	1611	1079	329	266


	Down- regulated	616	587	298	89	53

*ETC-specific DEGs were annotated using Mapman and KEGG pathway analysis. Those regarded central to ETC *trans*-differentiation and function were grouped as functional DEGs (Supplementary Table S7). ETC-specific DEGs were determined using limmaR from six replicate batches of cotyledons for adaxial epidermal cells and edgeR from three replicate batches of cotyledons for storage parenchyma cells. Signal-sensitive ETC-specific DEGs, that is, those regulated by known signals, were identified as those whose RPKM values were found to be significantly altered by signal blockers (*p* ≤ 0.05). Statistical significance determined from six replicate batches of control cotyledons and three replicate batches of cotyledons cultured on signal blockers using an unpaired *t*-test*.

#### RT-qPCR Validation of RNA-Seq Expression Data

Gene expression profiles obtained using RNA-seq were validated by RT-PCR (**Figure 1**). Transcripts of 14 genes encoding proteins linked with the four identified signals were selected. Of these, nine were UWL-specific (six up-regulated, three down-regulated) and five WI papillae specific (three up-regulated, two down-regulated). RT-qPCR estimates of gene expression were normalized as Log_2_ fold change against their 0 h expression levels and plotted against the corresponding RNA-seq data. When fitted to a linear regression analysis the two estimates of gene expression were highly correlated indicating that the DEG estimates were reliable (Supplementary Figure S4).

#### DEGs in Key Functional Categories Central to TC *Trans*-Differentiation and Nutrient Transport

Genes encoding proteins participating in signaling, intracellular organization and cell wall construction are considered central to TC *trans*-differentiation while nutrient transporters are core to TC function (**Figure 1**). Genes were assigned to these categories based on biochemical and enzymatic functions as annotated by Mapman Mercator and KEGG (Supplementary Table S7). Mapman and KEGG annotations have been effectively used to investigate genes encoding proteins contributing to mycorrhizal associations (Calabrese et al., 2017) and root hair growth (Damiani et al., 2016).

Inclusion of Mapman and KEGG categories into each functional category was informed by previously reported transcriptome analyses of developing ETCs (Zhang et al., 2015d). Thus, the signaling category was allocated genes encoding proteins for biosynthesis, action and metabolism of hormones, respiratory burst oxidases, superoxide dismutases and peroxidases as well as Ca^2+^ transporters and proteins participating in Ca^2+^ signaling pathways (Andriunas et al., 2013; Thiel, 2014; Zhang et al., 2015a). Map kinases were added on the basis that these act as messengers for H_2_O_2_ and Ca^2+^ signaling in plant cells (e.g., Wurzinger et al., 2011; Meng and Zhang, 2013). The intracellular organization category was ascribed genes encoding proteins contributing to microdomain formation in plasma membranes (Thiel, 2014), cytoskeleton and vesicle trafficking (Thiel, 2014; Zhang et al., 2015b, 2017). The cell wall category captured genes encoding proteins involved in the biosynthesis, modification and degradation of cell walls (Thiel, 2014; Zhang et al., 2015d). The central role TCs play in nutrient transport (Offler et al., 2003) justified a category covering genes encoding nutrient transporters (**Figure 1**).

#### Strategies to Assess Impact of Signaling Molecules on Expression of ETC-Specific DEGs

How key the signals, auxin, ethylene, H_2_O_2_ and Ca^2+^, impacted gene expression during wall labyrinth construction was assessed by performing RNA-seq on ETCs isolated from cotyledons cultured in the presence of their pharmacological inhibitors (**Figure 1** and Supplementary Tables S2, S3). The analysis was confined to control ETC-specific DEGs in the four functional gene categories (Supplementary Table S7) across each temporal expression profile (UWL-, WI papillae-specific and shared across both phases of wall labyrinth construction –**Figure 1**). Impacts of pharmacological removal of each signal were detected by determining whether the agent caused a statistically significant change of the control RPKM value of each DEG using an unpaired *t*-test. These tests were carried out on 3-h RPKM data for the UWL-specific DEGs and on 12-h RPKM data for WI papillae-specific and UWL/WI papillae shared DEGs (Supplementary Figure S5).

### Overall Spatiotemporal Expression Profiles of ETC DEGs

RNA-seq analysis detected 33,423 and 27,437 expressed unigenes in the epidermal/ETCs and SPCs of cultured *V*. *faba* cotyledons respectively (Supplementary Table S8). As reported by Zhang et al. (2015d), numbers of unigenes expressed at the two phases of wall labyrinth construction in *trans*-differentiating ETCs were temporally stable (Supplementary Table S8). A similar temporal profile was detected in nematode induced giant cells of rice roots (Ji et al., 2013). The 12–16% higher number of unigenes detected in the epidermal and ETCs compared to SPCs (Supplementary Table S8) reflects the fact that the reference library used to map expressed genes was generated from peels of epidermal cells and developing ETCs (Zhang et al., 2015d).

The total number of DEGs detected in the ETCs and underlying SPCs during TC *trans*-differentiation were 10,676 and 3,772 respectively (Supplementary Table S6). Thus 30% of the unigenes expressed in the ETCs underwent significant change compared to 14% in the SPCs (cf. Supplementary Table S8). For the ETCs, the proportion of DEGs was higher than the 22% recorded for nematode-induced giant cells of rice roots across two phases of their wall labyrinth development (Ji et al., 2013). Comparable DEG percentages have been recorded for other single cell transcriptomes including those of tip growing pollen tubes (Zhao et al., 2016) and root hairs (Hey et al., 2017).

Consistent with a rapid shift in cell fate, 67% of ETC DEGs were detected during the first 3 h of *trans*-differentiation to a TC-morphology with comparable numbers of up- and down-regulated DEGs. During the later stages of *trans*-differentiation, the ratio of up- to down-regulated DEGs increased two to three-fold (**Table 1**). Such a developmental change in the ratio of up- to down-regulated DEGs also occurs during *trans*-differentiation of cucumber fruit epidermal cells into trichomes (Chen et al., 2014).

A 2.8-fold higher DEG number in the ETCs compared to SPCs is consistent with the former undergoing *trans*-differentiation from an epidermal to ETC fate while the SPCs remain committed to a SPC fate. This is further reflected by the differential in ratios between up- and down-regulated DEGs for the two cell types with a 2.3-fold higher ratio exhibited by the SPCs. This differential is most pronounced during the first 3 h of ETC induction. Here the ratio is 1.1 and 5.1 for the ETCs and SPCs respectively indicating a large cohort of epidermal cell fate genes were switched off as the epidermal cells transition to an ETC fate. Thereafter, the ratios between the cell types fell into the range of two to three (Supplementary Table S6). Similar ratios of up- and down-regulated genes were detected in nematode-induced TC giant cells of tomato roots at 7 days post infection (Portillo et al., 2013). In contrast, nematode-induced TC giant cells of rice roots at the stage of WI papillae formation, strongly favored up-regulated genes (Ji et al., 2013). The observed differences are suggested to be more apparent than real. They likely reflect the rapid (h) temporal changes in gene expression found for ETCs. For example, transcript levels of ethylene and ROS biosynthetic genes undergo marked changes within the first 3 h of cotyledon culture (Zhou et al., 2010; Andriunas et al., 2012).

Of the total DEGs expressed in the *trans*-differentiating ETCs, a high proportion (91%) representing 9,742 DEGS, were found to be ETC specific (**Table 1**). This indicates that ETC-centric differences in quantitative gene expression profiles between ETCs and SPCs contribute significantly to driving ETC *trans*-differentiation (Supplementary Table S6). Of the 579 DEGs shared between the ETCs and SPCs, up-regulated DEGs across 0 – 3 h or 3 – 12 h exhibited the higher proportion (12 and 16% respectively) compared to 3 and 2% for down-regulated DEGs across these phases of wall labyrinth construction (Supplementary Table S6).

### Profiling ETC-Specific DEGS Within Each TC Functional Category

The TC-related functional categories of expressed genes chosen (Supplementary Table S7) represent 26% of the annotated ETC-specific DEG population with this proportion evenly represented by up- and down-regulated DEGs across both phases of wall labyrinth construction (**Table 1**). This proportion is somewhat higher than that found for ETC-specific expression of unigenes allocated to these functional categories (i.e., 17% – Zhang et al., 2015d). However, consistent with total DEG numbers, those found to be ETC-specific (i.e., 1,371; **Table 1**) were 3.8-fold greater than those of ETC-specific transcripts (365) reported in Zhang et al. (2015d). This finding further highlights the role DEGs play in regulating developmental pathways underpinning *trans*-differentiation to a TC phenotype.

The principal categories accounting for the remaining 74% of ETC-specific DEGs were general transcriptional regulation (21%, Mapman bins ‘RNA,’ ‘DNA’), general protein biosynthesis and modification (19%, Mapman bin ‘protein’), miscellaneous cell function and development (13%, Mapman bins ‘misc,’ ‘cell,’ ‘development’), general metabolism (12%, Mapman bins ‘major CHO metabolism,’ ‘minor CHO metabolism,’ ‘lipid metabolism,’ ‘amino acid metabolism’ and ‘secondary metabolism’) and stress response (9%, Mapman bin ‘stress’; for more details, see Supplementary Table S9).

**FIGURE 2:**
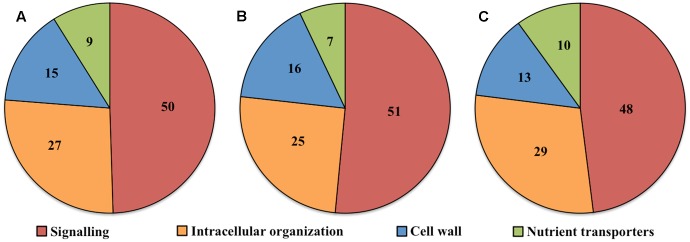
Pie charts showing the proportion (%) of **(A)** total, **(B)** up-regulated and **(C)** down-regulated ETC-specific DEGs in each key TC functional category (signaling, intracellular organization, cell wall, nutrient transporters) during wall labyrinth construction. DEGs in each functional category were classified based on their annotation in Mapman Mercator and KEGG functional analysis (see Supplementary Table S7).

Transcript numbers of ETC-specific DEGs detected within each functional category were in descending order signaling, intracellular organization, cell wall and nutrient transporters. These respectively accounted for 50, 27, 15, and 9% of the total ETC-specific DEG numbers in the four functional TC categories (**Figure 2A**). Up- and down-regulated DEG numbers also reflected the broad trend of DEG allocation between functional categories (**Figures 2B,C**). This finding indicated a substantive relative investment into DEGs encoding proteins participating in signaling pathways to orchestrate *trans*-differentiation to a TC phenotype. A similar proportionate investment into signaling was noted for expression of ETC-specific genes (Zhang et al., 2015d). However, in contrast to the disproportionate allocation of ETC-specific DEGs across the remaining functional categories, comparable portions of transcript numbers of ETC-specific genes were detected in groups encoding proteins involved in intracellular organization, cell wall and nutrient transporters (Zhang et al., 2015d). This suggests that ETC-specific DEGs play a greater role in the transcriptional regulation of genes in these functional categories compared to those encoding proteins with a signaling function.

Ratios of DEGs encoding proteins involved in signaling compared to cell wall building and nutrient transporters, 3.5X and 5.5X respectively (**Table 2**), were similar to those reported for *trans*-differentiating root cells penetrated by hyphae of mycorrhizal fungi (Calabrese et al., 2017). In contrast, investment into expression of genes encoding intracellular organization proteins was relatively higher in the ETCs, highlighting the complexity of building their wall labyrinth. A significant decrease of 67% in up-regulated DEGs involved in signaling specific for UWL versus WI papillae formation fits with the rapid change in cell fate during the former phase of ETC development (**Table 2**). However, an 80% decrease in up-regulated DEGs encoding intracellular organization proteins is more difficult to reconcile with a perceived greater complexity in constructing WI papillae compared to depositing the UWL (**Table 2**). A possible explanation could be that a significant portion of genes in this functional category are transcribed during UWL formation and are translated or post-translationally activated during WI papillae formation (e.g., see Zhang et al., 2017).

**Table 2:** Numbers of ETC-specific DEGs, within each TC functional category, responsive to one or a combination of ETC regulatory signals during the two phases of wall labyrinth construction (*p* ≤ 0.05).

Temporal expression profile	DEG regulation	Signaling			Intracellular organization			Cell wall			Nutrient transporters		
		Total DEGs	DEGs regulated by:		Total DEGs	DEGs regulated by:		Total DEGs	DEGs regulated by:		Total DEGs	DEGs regulated by:	
			One signal	Multiple signals		One signal	Multiple signals		One signal	Multiple signals		One signal	Multiple signals
UWL specific	Up	203	71	56	112	47	27	65	29	15	32	13	7
	Down	163	60	48	107	36	18	53	16	18	46	17	14
WI specific	Up	67	20	31	23	6	9	26	6	16	14	3	7
	Down	23	5	11	16	6	6	2	2	0	1	0	1
Shared UWL/WI	Up	178	45	105	87	25	40	45	12	21	19	6	12
	Down	52	14	21	21	2	8	11	2	4	5	0	2

### Global Evaluation of ETC-Specific DEGs Regulated by Signals within each Functional TC Category

Expression levels of DEGs regulated by one or more of the four known signals, auxin, ethylene, H_2_O_2_ (Andriunas et al., 2013) and Ca^2+^ (Zhang et al., 2015a), accounted for 69% of the ETC-specific DEGs associated with the four functional gene categories linked with ETC development and membrane transport of nutrients (**Table 1**). The greater proportions of ETC-specific DEGs acted upon by the known signals were those up- and down-regulated specifically during WI papillae formation and for those up-regulated across both phases of wall labyrinth formation (**Table 1**).

Signaling molecules, yet to be confirmed, control expression levels of the remaining 31% of the ETC-specific functional categories of DEGs (**Table 1**). Additional signaling candidates that are expressed during ETC development are ABA and gibberellins (Zhang et al., 2015d). Other signaling candidates suggested to regulate wall labyrinth construction in other cell systems include jasmonic acid shown to influence phloem parenchyma TC development in Arabidopsis leaves (Amiard et al., 2007) and cytokinins driving basal endosperm TC development in maize caryopses (Yuan et al., 2016). These signaling candidates are being explored in another study.

**FIGURE 3:**
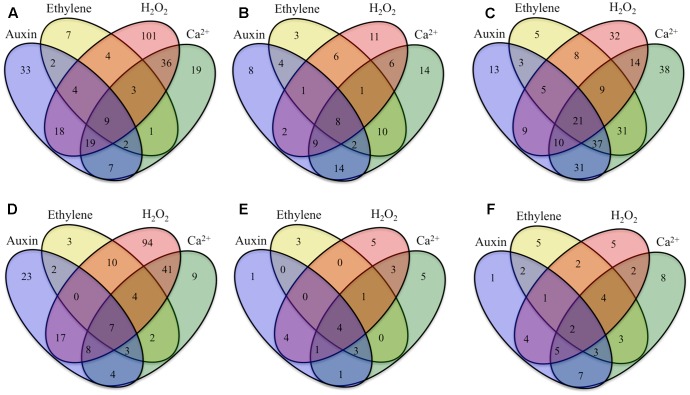
Venn diagrams showing numbers of ETC-specific DEGs in key TC functional categories (signaling, intracellular organization, cell wall, nutrient transporters), sensitive to signaling molecules known to regulate ETC *trans*-differentiation (auxin, ethylene, H_2_O_2_ and Ca^2+^) that were **(A–C)** up-regulated or **(D–F)** down-regulated specifically during construction of **(A,D)** UWL, **(B,E)** WI papillae, or **(C,F)** shared between UWL and WI papillae construction phases. Signal-sensitive ETC-specific DEGs were identified as those whose RPKM values were found to be significantly altered by signal blockers (*p* ≤ 0.05). Statistical significance was determined from six replicate batches of control cotyledons and three replicate batches of cotyledons cultured on signaling blockers using an unpaired *t*-test.

**FIGURE 4:**
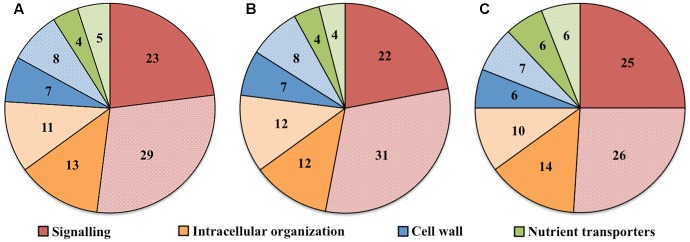
Pie charts showing the proportion (%) of **(A)** total number, **(B)** up-regulated or **(C)** down-regulated ETC-specific DEGs in key TC functional categories (signaling, intracellular organization, cell wall, nutrient transporters) whose change in expression was regulated by one (dark color fill) or multiple (light color fill) signaling molecules (auxin, ethylene, H_2_O_2_ and Ca^2+^) known to regulate ETC *trans*-differentiation during wall labyrinth construction.

To explore the relationship between the cohort size of ETC-specific DEGs regulated by one or various combinations of the signals known to orchestrate wall labyrinth assembly, Venn diagrams were constructed for the number of ETC-specific DEGs linked with the four TC functional categories for each temporal transcript profile (**Figure 3** and Supplementary Figures S6–S9). Overall, the influence of the signals acting alone (48%) or in combination (52%) on expression levels of ETC-specific DEGs was shared equally across the four functional TC categories (**Figure 4** and **Table 2**). However, there was a shift in dominance as wall labyrinth construction proceeded from DEGs being regulated by one signal during UWL formation (60%) to combinations of signals exerting an influence during WI papillae assembly (58%; **Table 2**). The structural complexity of assembling WI papillae extending vertically into the cytoplasm from loci on the UWL is consistent with a need for an increased sophistication of the signaling network regulating such a complex form of cell wall construction. Consistent with this interpretation, the cell wall functional category was impacted the most by signal combinations (72%; **Table 2**).

### Cohorts of ETC-Specific TC Functional DEGs Regulated by Signals Acting Alone

For those ETC-specific DEGs, within the specified TC functional categories, responsive to one of the four signals (**Figure 4** and **Table 2**), the data demonstrated that, across wall labyrinth formation, the descending order of regulated DEG numbers was H_2_O_2_ (56%), Ca^2+^ (21%), auxin (18%) and ethylene (5%; **Figure 3**). Domination by H_2_O_2_ applied to up- and down-regulated DEGs in all functional TC categories (**Table 3**; Supplementary Figures S6–S9) and was most pronounced during UWL formation (**Figures 3A,D**). This observation is consistent with the central role played by H_2_O_2_ in initiating and directing cell wall deposition to the outer planar cell wall domain of developing ETCs to construct the wall labyrinth (Andriunas et al., 2012; Xia et al., 2012). The significant role played by H_2_O_2_ in regulating the transcriptome during ETC *trans*-differentiation contrasts with auxin and ethylene functioning as master transcriptional regulatory molecules in root hairs (Velasquez et al., 2016) and trichomes (Zhao et al., 2015a,b). Never-the-less, in other systems ROS plays a core regulatory role in initiating substantive transcriptional cascades for stress response and developmental pathways (Willems et al., 2016). In the context of the present study, H_2_O_2_ has been shown to exert a significant regulatory influence over expression of genes encoding cellulose biosynthesis in elongating root hairs (Nestler et al., 2014) and of plant hormonal and signal transduction pathways, as well as cell wall modifying enzymes during H_2_O_2_-induced adventitious rooting (Li et al., 2017). Roles for ROS in controlling expression of genes encoding proteins forming structural elements, or controlling the dynamics, of the plant cytoskeleton as well as membrane transporters of nutrients are less well known. The influence of H_2_O_2_ in regulating ETC-specific DEGs decreased as ETC development transitioned from UWL to WI papillae formation to a shared role with Ca^2+^ (**Figure 3**) in the signaling, intracellular organization and nutrient transport functional categories (Supplementary Figures S6, S7, S9). In contrast, Ca^2+^ controlled two-fold more DEGs in the cell wall functional category (Supplementary Figure S8).

**Table 3:** Numbers of ETC-specific DEGs, within each TC functional category, responsive to each ETC regulatory signal across wall labyrinth construction (*p* ≤ 0.05).

Signal	Signaling		Intracellular organization		Cell wall		Nutrient transporters		Total		Grand total
	Up	Down	Up	Down	Up	Down	Up	Down	Up	Down	
Auxin	27	9	18	10	4	2	5	3	54	24	78
Ethylene	5	4	3	4	3	2	0	1	11	11	22
H_2_O_2_	63	53	42	22	27	15	12	14	144	104	248
Ca^2+^	37	13	15	8	13	1	6	0	71	22	93

Auxin and Ca^2+^ were the second ranked influential signals acting alone to regulate expression of ETC-specific DEGs (**Table 2**). Across ETC development Ca^2+^ exerted greater control over the signaling and cell wall TC functional categories while auxin regulated a slightly higher number of ETC-specific DEGs encoding proteins involved in intracellular organization and nutrient transporter categories (**Table 3**). In addition to the well described Ca^2+^ signaling pathways regulating plant development (Niu and Liao, 2016), a recent study has documented expression of genes encoding cell wall metabolizing enzymes positively regulated by Ca^2+^ along with similar impacts on genes encoding nutrient transporters (Wang et al., 2015). Similarly, as reported for other plant systems, auxin regulates expression of genes encoding proteins contributing to intracellular organization and to membrane transport of nutrients (e.g., Dai et al., 2009; Omelyanchuk et al., 2017). During UWL formation, two-fold more ETC-specific DEGs were regulated by auxin compared to Ca^2+^ (**Figures 3A,D**); an effect evident across all TC functional categories (Supplementary Figures S6–S9). Comparable to the role played by auxin in root hair initiation (Velasquez et al., 2016), this gene expression profile is consistent with auxin being positioned as an upstream component in the signaling cascade regulating ETC *trans*-differentiation (Zhou et al., 2010). The relative contributions of auxin and Ca^2+^ reversed once WI papillae formation commenced (**Figures 3B,E**; Supplementary Figures S6–S9). Consistent with WI papillae assembly being Ca^2+^-regulated (Zhang et al., 2015a), Ca^2+^ impacted 75% of the WI-specific DEGs in the cell wall functional category with the remainder being comprised of ethylene-sensitive DEGs (Supplementary Figure S8).

Ethylene acting alone is the least influential signal affecting transcription of ETC-specific DEGs across all functional categories. This feature is most pronounced for genes encoding nutrient transporters where only one down-regulated DEG specifically expressed during UWL formation was found to be ethylene sensitive (**Table 3** and Supplementary Figure S9). However, ethylene exerted a four-fold greater influence than auxin on expression of genes in the cell wall functional category as the wall labyrinth transitioned from UWL to WI papillae construction (Supplementary Figure S8) indicating a possible ethylene regulation of WI papillae deposition (Andriunas et al., 2012). A similar conclusion can be reached for auxin since ETC-specific DEGs, sensitive to auxin, were detected during this phase of wall labyrinth construction in all TC functional categories (**Figures 3C,F** and Supplementary Figures S6–S9). We tested this claim by blocking auxin and ethylene signaling following cessation of UWL deposition by culturing cotyledons on media containing pharmacological inhibitors of both hormones. The auxin block halted on-going development of WI papillae while that for ethylene elicited a partial inhibition (Supplementary Table S10). The latter response is consistent with ethylene regulating expression of Ca^2+^-permeable channels responsible for forming plumes of elevated [Ca^2+^]_cyt_ defining loci for WI papillae formation (Zhang et al., 2015c). The greater influence of auxin over ethylene on WI-specific DEG expression (Supplementary Figures S6–S9) and on WI papillae formation (Supplementary Table S10) is not readily reconciled with the proposed linear signaling cascade regulating wall labyrinth assembly with ethylene positioned downstream of auxin (Zhou et al., 2010). Together, the observations reported above suggest that auxin likely acts at more than one point in the signaling cascade regulating wall labyrinth development.

### ETC-Specific DEGs within Each Functional TC Category Regulated by Signal Combinations

All 11 combinations of the four signals affected expression levels of a cohort of ETC-specific DEGs encoding transcripts that fall into the four specified functional categories across wall labyrinth formation (**Figure 3**). The exceptions to this generalization were an absence of up- and down-regulated DEGs sensitive to the combination of auxin/ethylene/H_2_O_2_ and of down-regulated DEGs sensitive to auxin/ethylene, auxin/ethylene/Ca^2+^, auxin/ethylene/H_2_O_2_/Ca^2+^, ethylene/Ca^2+^ and ethylene/H_2_O_2_/Ca^2+^ in some of the functional categories (**Table 4**). Of the eleven signal combinations, seven exerted predominant control accounting for 84% of DEGs regulated by all possible signal combinations. The standout was the H_2_O_2_/Ca^2+^ combination affecting expression levels of approximately two-fold more ETC-specific DEGs than each of the remaining six combinations impacting a range of 47–63 ETC-specific DEGs per combination (**Table 4**). Significantly, either or both H_2_O_2_ and Ca^2+^ were present in all the latter signal combinations with Ca^2+^ acting on a greater portion of up-regulated ETC-specific DEGs compared to H_2_O_2_ (**Table 4**). Overall these findings highlight the central role played by H_2_O_2_ and Ca^2+^ signals in regulating gene expression of proteins orchestrating wall labyrinth formation and nutrient transporters (Andriunas et al., 2012; Zhang et al., 2015a). Cross talk between H_2_O_2_ and Ca^2+^ signaling has been identified in a number of plant developmental programs (Niu and Liao, 2016) including polarized tip growth of pollen tubes and root hairs (Mangano et al., 2016). The remaining four signal combinations, which affected the least number of ETC-specific DEGS (11–30), had ethylene as one of the partners (**Table 4**). However, Ca^2+^ offset the negative influence of ethylene except when H_2_O_2_ was present in the absence of auxin.

**Table 4:** Numbers of ETC-specific DEGs, within each TC functional category, responsive to multiple combinations of ETC regulatory signals across wall labyrinth construction (*p* ≤ 0.05).

Signal Combinations	Signaling		Intracellular organization		Cell wall		Nutrient transporters		Total		Grand total
	Up	Down	Up	Down	Up	Down	Up	Down	Up	Down	
Auxin/ethylene	5	3	2	1	1	0	1	0	9	4	13
Auxin/H_2_O_2_	16	10	6	10	5	3	2	2	29	25	54
Auxin/Ca^2+^	33	8	7	2	8	1	3	1	51	12	63
Auxin/ethylene/H_2_O_2_	2	0	6	1	0	0	2	0	10	1	11
Auxin/ethylene/Ca^2+^	27	6	6	0	4	2	3	1	40	9	49
Auxin/ H_2_O_2_/Ca^2+^	19	9	9	1	4	3	6	1	38	14	52
Auxin/ethylene/ H_2_O_2_/Ca^2+^	19	9	9	2	9	2	1	0	38	13	51
Ethylene/H_2_O_2_	7	6	5	3	5	2	1	1	18	12	30
Ethylene/Ca^2+^	30	1	7	1	3	3	2	0	42	5	47
Ethylene/ H_2_O_2_/Ca^2+^	6	5	4	2	3	1	0	2	13	10	23
H_2_O_2_/Ca^2+^	28	23	15	9	8	5	5	9	56	46	102

The global impacts of the various signal combinations described above were manifested in the expression responses of the signaling functional category of genes (**Table 4**) that contained the largest number of ETC-specific DEGs regulated by signal combinations (**Table 2**). Except for the dominance displayed by the H_2_O_2_/Ca^2+^ combination, the proportions of ETC-specific DEG numbers responding to the various signal combinations departed from the overall pattern and were functional category centric (**Table 4**). For instance, in the intracellular organization function category, auxin/H_2_O_2_ elicited the second strongest response and auxin/ethylene the least, with remaining signal combinations affecting a narrow range (6–10) of ETC-specific DEG numbers. In contrast, for the cell wall functional category, comparable responses were shared across all combinations except for auxin/ethylene and auxin/ethylene/H_2_O_2_ that affected proportionally lower numbers of ETC-specific DEGs (**Table 4**).

## Conclusion

An experimental design employing culture-induced *trans*-differentiating ETCs of *V. faba* cotyledons, exposed to media in the presence or absence of pharmacological blockers of signals known to regulate ETC *trans*-differentiation, identified an assemblage of ETC-specific DEGs within functional categories of genes encoding proteins considered responsible for wall labyrinth construction and TC function of nutrient transport. Significant alterations in transcript levels of these ETC-specific DEGs to pharmacological blockers of the known signals revealed that H_2_O_2_ acting alone, and in combination with Ca^2+^, controlled expression of the largest cohort (37% of the total) in the four TC functional gene categories across the two phases of wall labyrinth construction. Collectively these data offer a powerful resource to elucidate signal transduction pathways regulating expression of genes encoding proteins involved in intracellular organization and cell wall construction to assemble the wall labyrinth and transporters responsible for nutrient transport.

## Data Deposition

The cDNA sequence datasets of raw reads and assembled reference transcriptome library (Zhang et al., 2015d) supporting this article are available in the repository of the European Nucleotide Archive (ENA, https://www.ebi.ac.uk/ena) with the ENA accession number: PRJEB8906. Lists of ETC-specific DEGs in each functional category and the impact of pharmacological blockers of known signals on their expression are provided in Supplementary Table S11.

## Author Contributions

JP and CO conceived and designed the research project. H-MZ, SW, and XX performed the experiments and compiled the data sets. H-MZ assisted by KC and JP analyzed the data. H-MZ wrote the first draft of the manuscript that was revised by JP and reviewed by CO, KC, SW, XX, and H-MZ.

## Conflict of Interest Statement

The authors declare that the research was conducted in the absence of any commercial or financial relationships that could be construed as a potential conflict of interest.

## Footnotes

1 http://mapman.gabipd.org/web/guest/mercator

2 http://www.uniprot.org/

3 https://www.ncbi.nlm.nih.gov/COG/

4 http://www.genome.jp/kegg/

## Abbreviations

AA: ascorbic acid
AVG: aminoethoxyvinylglycine
BAPTA 1: 2-bis(*o*-aminophenoxy)ethane-N,N,N′,N′-tetraacetic acid
CDD: Conserved Domain Database
COG: Clusters of Orthologous Groups
DEG: differentially expressed gene
ETC: epidermal transfer cell
FDR: false discovery rate
H_2_O_2_: hydrogen peroxide
KEGG: Kyoto Encyclopedia of Genes and Genomes
MS: Murashige and Skoog
PCIB: *p*-chlorophenoxyisobutyric acid
RNA-seq: RNA-sequencing
ROS: reactive oxygen species
RPKM: reads per kilo base per million reads
SPC: storage parenchyma cell
TC: transfer cell
UWL: uniform wall layer
WI: wall ingrowth.
