# Supplementary material for: Transcript Profiling Identifies Gene Cohorts Controlled by Each Signal Regulating Trans-Differentiation of Epidermal Cells of Vicia faba Cotyledons to a Transfer Cell Phenotype
Source: Front Plant Sci. 2017 Nov 28;8:2021. doi: 10.3389/fpls.2017.02021 (PMC5712318; doi:10.3389/fpls.2017.02021)
Supplement: Supplementary file 1 [file Data_Sheet_1.ZIP › Supplementary files FF pdfs only/Supplementary Figure S1.pdf]

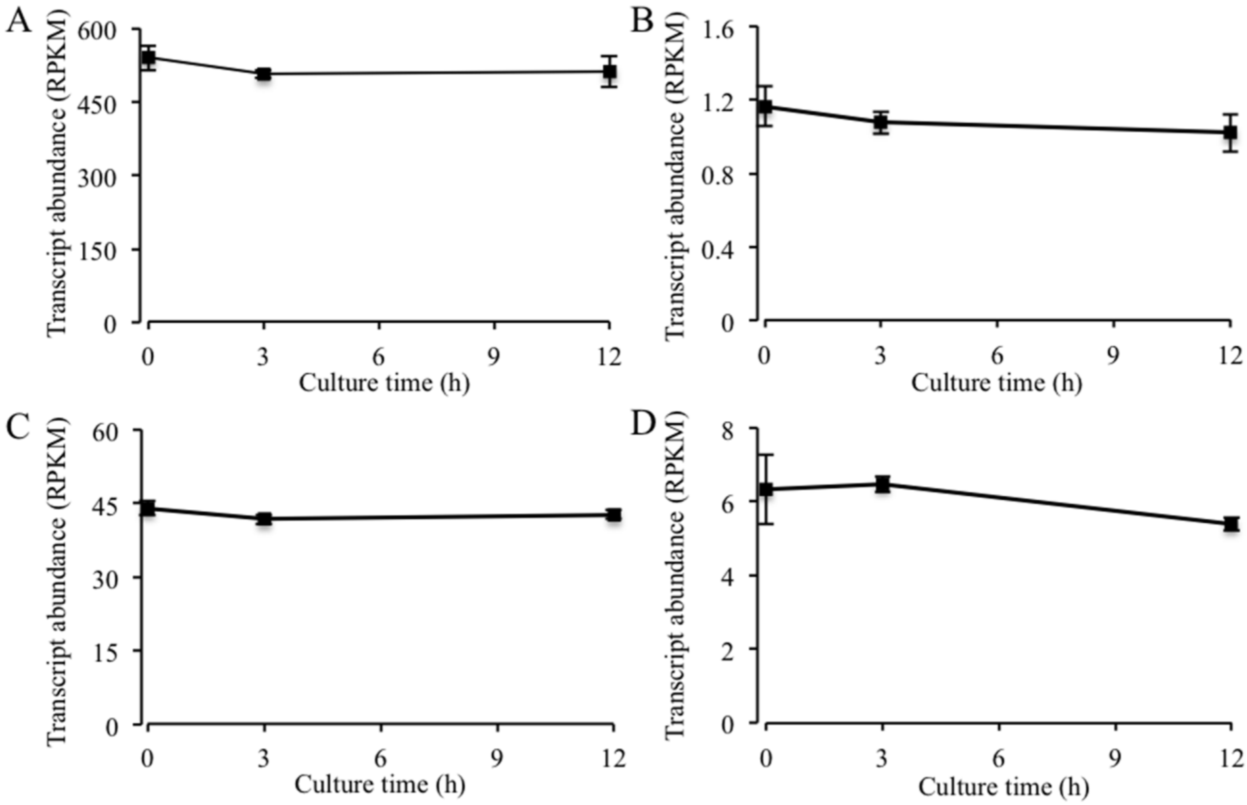

Supplementary Figure S1. Temporal expression profiles of housekeeping genes for normalization of real-time PCR studies of gene expression in adaxial epidermal cells of *V. faba* cotyledons cultured for specified times. (A) *Elongation factor 2-alpha (VfEF2α)*; (B) *NADH dehydrogenase subunit 4 (VfNADHD4)*; (C) *60S ribosomal protein subunit L2 (Vf60SL2)*; (D) *multi-domain cyclophilin type peptidyl-prolyl cis-trans isomerase G (VfPPlaseG)*. All candidates were further validated using real-time PCR to test their reliability as housekeeping genes (see Material and Methods for more details). Data are Means ± SEs of six replicate biological samples of adaxial epidermal cells (n = 6).
