# Supplementary material for: Transcript Profiling Identifies Gene Cohorts Controlled by Each Signal Regulating Trans-Differentiation of Epidermal Cells of Vicia faba Cotyledons to a Transfer Cell Phenotype
Source: Front Plant Sci. 2017 Nov 28;8:2021. doi: 10.3389/fpls.2017.02021 (PMC5712318; doi:10.3389/fpls.2017.02021)
Supplement: Supplementary file 1 [file Data_Sheet_1.ZIP › Supplementary files FF pdfs only/Supplementary Figure S2 .pdf]

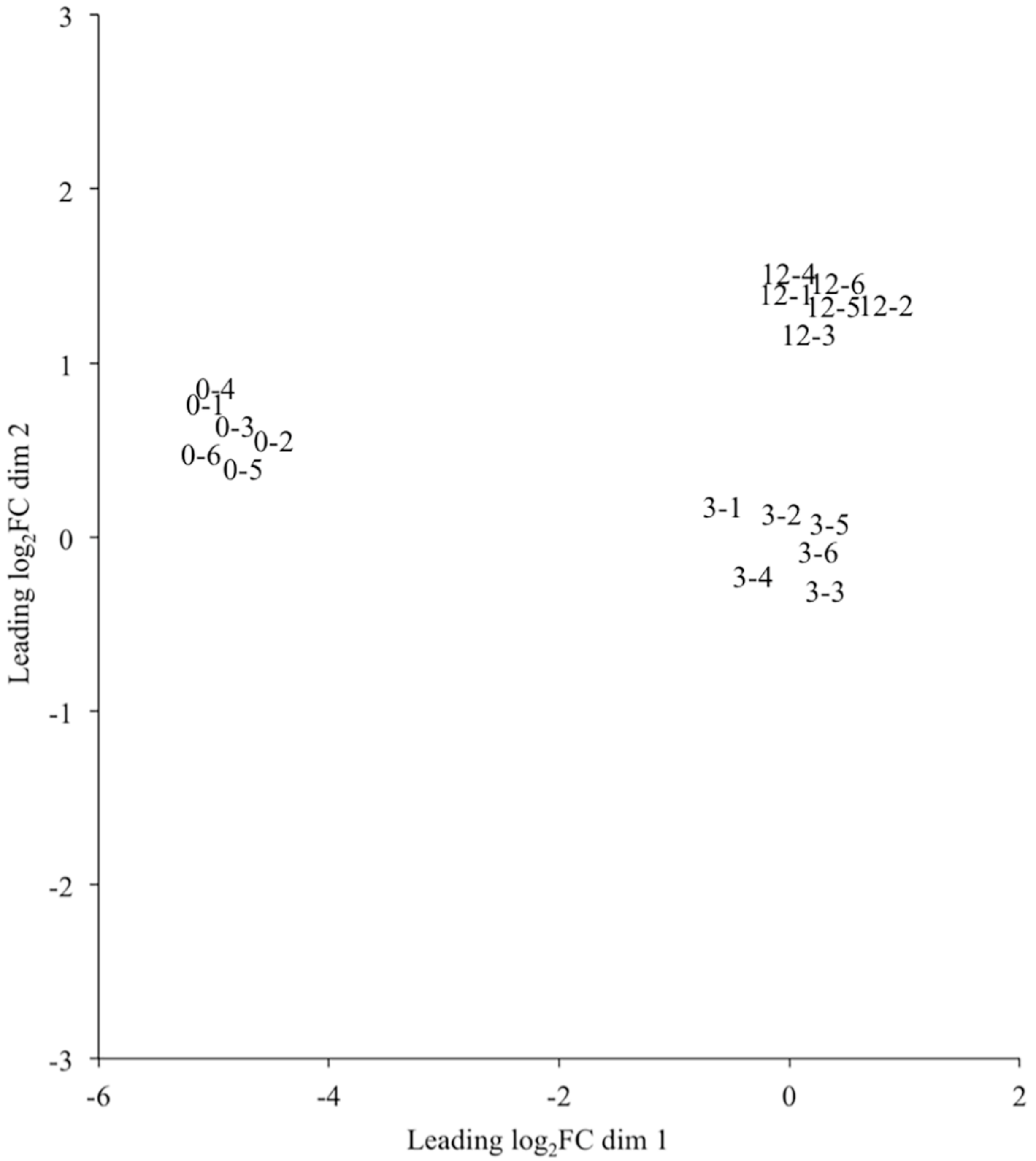

Supplementary Figure S2. Multi-dimensional scaling (MDS) plots of expression profiles of all unigenes in adaxial epidermal cells that were obtained from Zhang et al., 2015d (labelled as -1 to -3) and this study (labelled as reps - 4 to - 6). Epidermal cells were peeled from cotyledons that were freshly harvested (0 h, samples labelled 0 -) or cultured for 3 (3 -) or 12 (12 -) h in MS medium. The MDS was plotted using limmaR.
