# Supplementary material for: Transcript Profiling Identifies Gene Cohorts Controlled by Each Signal Regulating Trans-Differentiation of Epidermal Cells of Vicia faba Cotyledons to a Transfer Cell Phenotype
Source: Front Plant Sci. 2017 Nov 28;8:2021. doi: 10.3389/fpls.2017.02021 (PMC5712318; doi:10.3389/fpls.2017.02021)
Supplement: Supplementary file 1 [file Data_Sheet_1.ZIP › Supplementary files FF pdfs only/Supplementary Figure S3 docx.pdf]

Supplementary Figure S3. Temporal expression patterns (Log_2 fold change in RPKM in relation to T = 0 h) of differentially expressed genes (DEGs) in epidermal transfer cells (ETCs) and storage parenchyma cells (SPCs) of cultured *V. faba* cotyledons. Cotyledons were freshly harvested (0 h) or cultured in MS medium for 3 or 12 h before being processed for RNA-seq analysis. Temporal expression patterns identified between each sampling time were:

0 to 3 h up-regulated:

(a) 0 to 3 h Log_2 FC > 1, 3 to 12 h Log_2 FC < -1, -1 < 0 to 12 h Log_2 FC < 1

(b) 0 to 3 h Log_2 FC > 1, -1 < 3 to 12 h Log_2 FC < 1, -1 < 0 to 12 h Log_2 FC < 1

(c) 0 to 3 h Log_2 FC > 1, 3 to 12 h Log_2 FC < -1, 0 to 12 h Log_2 FC < -1.

0 to 3 h down-regulated:

(a) 0 to 3 h Log_2 FC < -1, 3 to 12 h Log_2 FC > 1, -1 < 0 to 12 h Log_2 FC < 1

(b) 0 to 3 h Log_2 FC < -1, -1 < 3 to 12 h Log_2 FC < 1, -1 < 0 to 12 h Log_2 FC < 1

(c) 0 to 3 h Log_2 FC < -1, 3 to 12 h Log_2 FC > 1, 0 to 12 h Log_2 FC > 1.

3 to 12 h up-regulated:

(a) -1 < 0 to 3 h Log_2 FC < 1, 3 to 12 h Log_2 FC >1 , 0 to 12 h Log_2 FC > 1

(b) -1< 0 to 3 h Log_2 FC < 1, -1 < 3 to 12 h Log_2 FC < 1, 0 to 12 h Log_2 FC > 1

(c) 0 to 3 h Log_2 FC < -1, 3 to 12 h Log_2 FC > 1, 0 to 12 h Log_2 FC > 1.

3 to 12 h down-regulated:

(a) -1 < 0 to 3 h Log_2 FC < 1, 3 to 12 h Log_2 FC < -1, 0 to 12 h Log_2 FC < -1

(b) -1< 0 to 3 h Log_2 FC < 1, -1 < 3 to 12 h Log_2 FC < 1, 0 to 12 h Log_2 FC < -1

(c) 0 to 3 h Log_2 FC > 1, 3 to 12 h Log_2 FC < -1, 0 to 12 h Log_2 FC < -1.

0 to 3 h and 3 to 12 h shared up-regulated:

(a) 0 to 3 h Log_2 FC > 1, 3 to 12 h Log_2 FC > 1, 0 to 12 h Log_2 FC > 1

(b) 0 to 3 h Log_2 FC > 1, -1 < 3 to 12 h Log_2 FC < 1, 0 to 12 h Log_2 FC > 1

(c) 0 to 3 h Log_2 FC > 1, 3 to 12 h Log_2 FC < -1, 0 to 12 h Log_2 FC > 1.

0 to 3 h and 3 to 12 h shared down-regulated:

(a) 0 to 3 h Log_2 FC < -1, 3 to 12 h Log_2 FC < -1, 0 to 12 h Log_2 FC < -1

(b) 0 to 3 h Log_2 FC < -1, -1 < 3 to 12 h Log_2 FC < 1, 0 to 12 h Log_2 FC < -1

(c) 0 to 3 h Log_2 FC < -1, 3 to 12 h Log_2 FC > 1, 0 to 12 h Log_2 FC < -1.

The expression profile of all DEGs within each pattern was summarized using Pearson's correlation as distance matrix and presented below. Expression data was generated from six replicate batches of cotyledons for adaxial epidermal cells (0 h) and ETCs (3 and 12 h) and from three replicate batches of cotyledons for SPCs (3 and 12 h). Only DEGs with a FDR corrected p ≤0.05 were retained.

* 0 to 3 h (c) up-regulated DEGs exhibit an identical temporal expression pattern to 3 to 12 h (c) down-regulated DEGs.

** 0 to 3 h (c) down-regulated DEG exhibit an identical temporal expression pattern to 3 to 12 h (c) up-regulated DEGs.

Temporal change during		Expression pattern	Number of up-regulated DEGs in:		Expression pattern	Number of down-regulated DEGs in:	
			ETC	SPC		ETC	SPC
	(a)	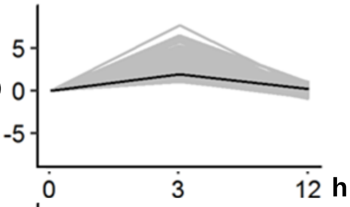	2516	809	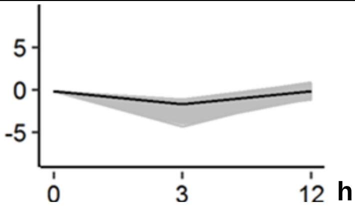	1698	117
0 to 3 h	(b)	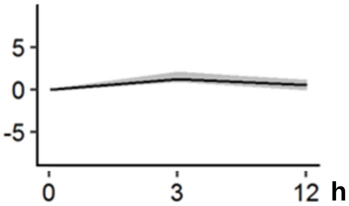	1293	949	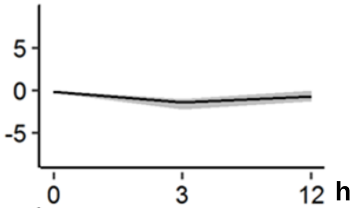	1696	228
	(c)	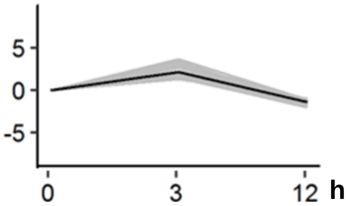	24^{*}	25^{*}	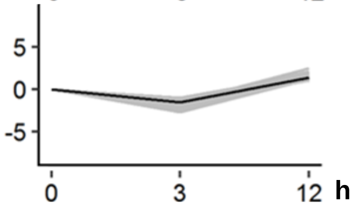	37^{**}	4^{**}
	(a)	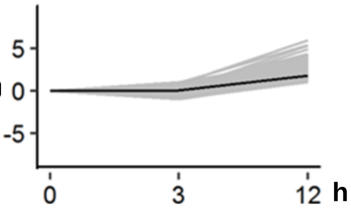	554	444	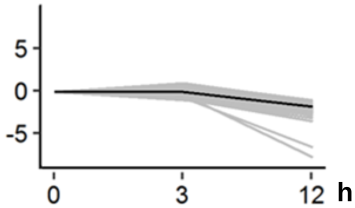	128	197
3 to 12 h	(b)	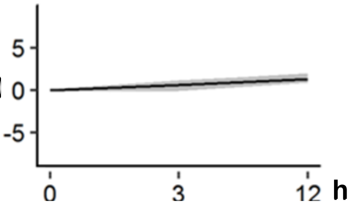	280	403	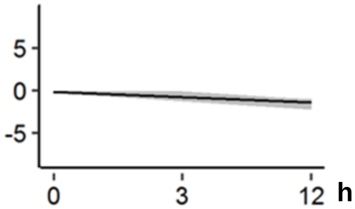	186	205
	(c)	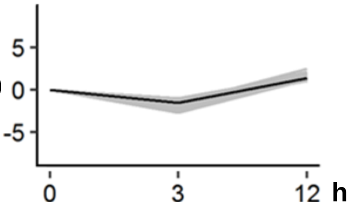	37^{**}	4^{**}	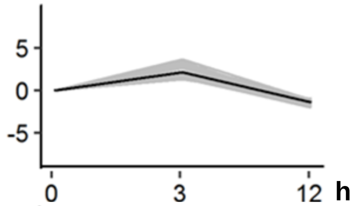	24^{*}	25^{*}
	(a)	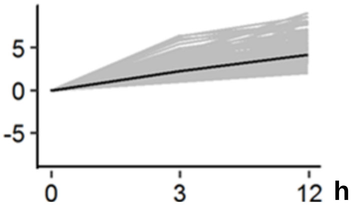	151	43	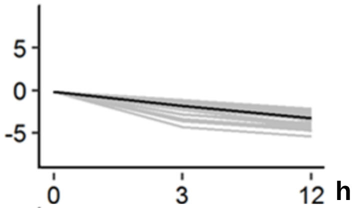	11	3
Shared 0 to 3 h 3 to 12 h	(b)	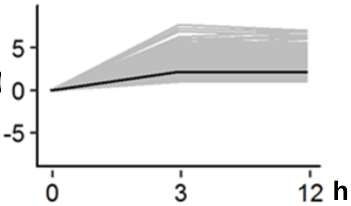	1023	248	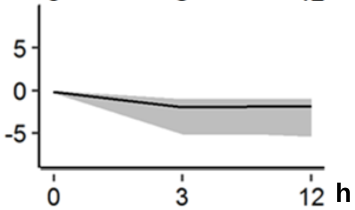	538	98
	(c)	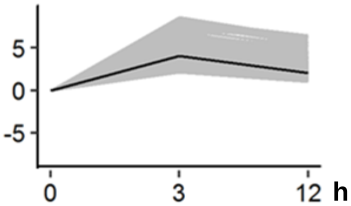	535	27	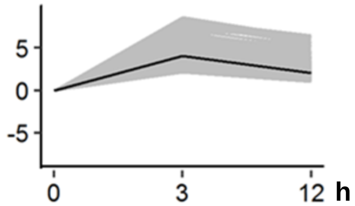	67	1
