# Supplementary material for: Transcript Profiling Identifies Gene Cohorts Controlled by Each Signal Regulating Trans-Differentiation of Epidermal Cells of Vicia faba Cotyledons to a Transfer Cell Phenotype
Source: Front Plant Sci. 2017 Nov 28;8:2021. doi: 10.3389/fpls.2017.02021 (PMC5712318; doi:10.3389/fpls.2017.02021)
Supplement: Supplementary file 1 [file Data_Sheet_1.ZIP › Supplementary files FF pdfs only/Supplementary Figure S4 .pdf]

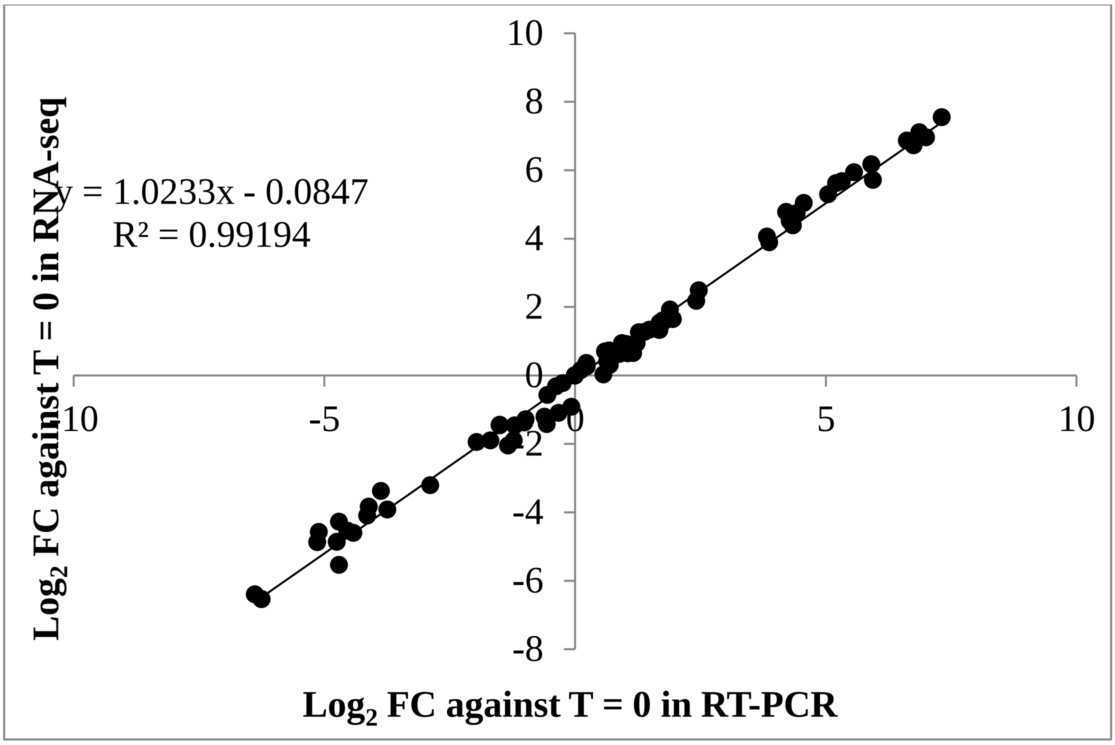

Supplementary Figure S4. Linear regression analysis of expression patterns of unigenes obtained from RNA-seq and quantitative RT-PCR. Fourteen unigenes were tested. These were: U12508 (flavin-containing monooxygenase YUCCA1), U24743 (tryptophan aminotransferase-like protein 2), CL6949C2 (auxin induced protein 5NG4), U23619 (putative 1-aminocyclopropane-1-carboxylate synthase), U919 (1-aminocyclopropane-1-carboxylate oxidase-like protein), U9449 (ethylene responsive factor), CL10622C1 (NADPH oxidase), U830 (peroxidase), U26632 (two pore calcium channel protein 1), U23718 (cyclic nucleotide-gated ion channel 2), U29158 (autoinhibited calcium ATPase), CL6498C2 (calmodulin binding protein), U14740 (pectin esterase) and U7484 (little zipper protein 4). For these unigenes, their expression values in freshly harvested cotyledons (0 h, all unigenes) and cotyledons cultured for 3 h (CL6498C2, CL6949C2, CL10622C1, U7484, U9449, U12508, U14740, U23619, U24743) or 12 h (U830, U919, U23718, U26632, U29153) in the absence (control) or presence of 200 µM PCIB, 100 µM AVG, 10 mM ascorbic acid, 600 µM BAPTA, were obtained. Log_2 fold changes in expression levels of all unigenes against those of freshly harvested cotyledons were calculated from RNA-seq and RT-PCR data sets. Data are log_2 fold change of mean expression levels (from three biological replicates) at specified culture times and treatments in relation to T = 0 h, N = 70 data comparisons.
