# Supplementary material for: Transcript Profiling Identifies Gene Cohorts Controlled by Each Signal Regulating Trans-Differentiation of Epidermal Cells of Vicia faba Cotyledons to a Transfer Cell Phenotype
Source: Front Plant Sci. 2017 Nov 28;8:2021. doi: 10.3389/fpls.2017.02021 (PMC5712318; doi:10.3389/fpls.2017.02021)
Supplement: Supplementary file 1 [file Data_Sheet_1.ZIP › Supplementary files FF pdfs only/Supplementary Figure S5 .pdf]

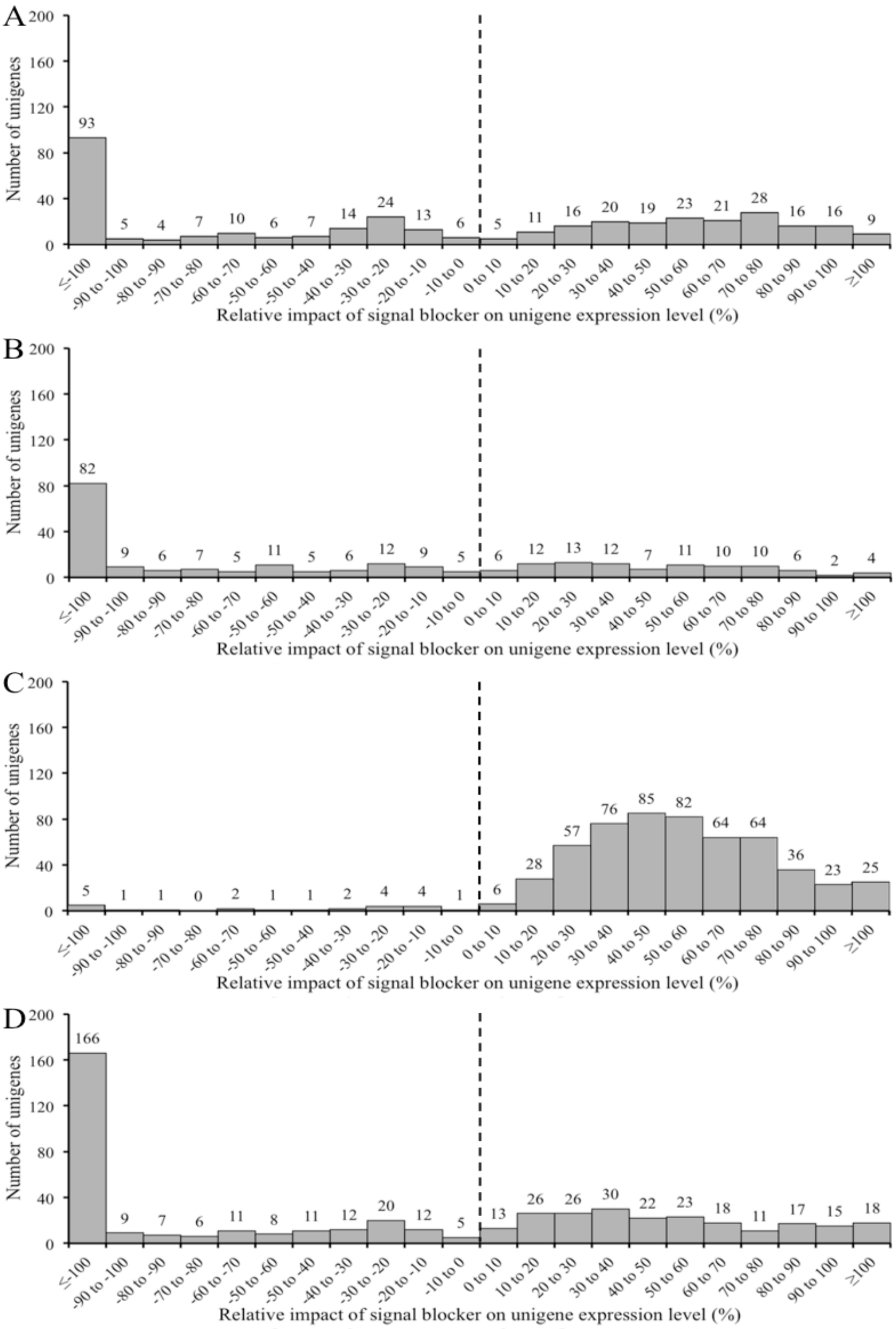

Supplementary Figure S5 Relative impacts of pharmacological blockers of signals, known to regulate ETC *trans*-differentiation, on expression levels of ETC-specific DEGs in TC functional categories. Cotyledons were freshly harvested or cultured in liquid MS medium in the absence/presence of: (A) auxin action inhibitor PCIB; (B) ethylene biosynthesis inhibitor, AVG; (C) extracellular ROS scavenger, ascorbic acid; (D) extracellular Ca^{2+} chelator, BAPTA. Significant impacts of pharmacological agents on DEG expression levels were determined by comparing mean expression levels (RPKM) of corresponding DEGs from cotyledons cultured in the presence versus absence of each agent using an unpaired, unequal sample size, two tailed t-test ($p \leq 0.05$). Impacts of signalling molecules on expression levels of DEGs were classified as positive (i.e. removal of signal significantly decreased RPKM value compared to that of the corresponding control value) or negative (removal of signal significantly increased expression levels of DEGs compared to that of the corresponding control). Expression data were generated from batches of cotyledons cultured in the absence (n = 6 batches) and presence (n = 3 batches) of the specified pharmacological signal blocker.
