# Supplementary material for: Transcript Profiling Identifies Gene Cohorts Controlled by Each Signal Regulating Trans-Differentiation of Epidermal Cells of Vicia faba Cotyledons to a Transfer Cell Phenotype
Source: Front Plant Sci. 2017 Nov 28;8:2021. doi: 10.3389/fpls.2017.02021 (PMC5712318; doi:10.3389/fpls.2017.02021)
Supplement: Supplementary file 1 [file Data_Sheet_1.ZIP › Supplementary files FF pdfs only/Supplementary Figures S6 - S9 .pdf]

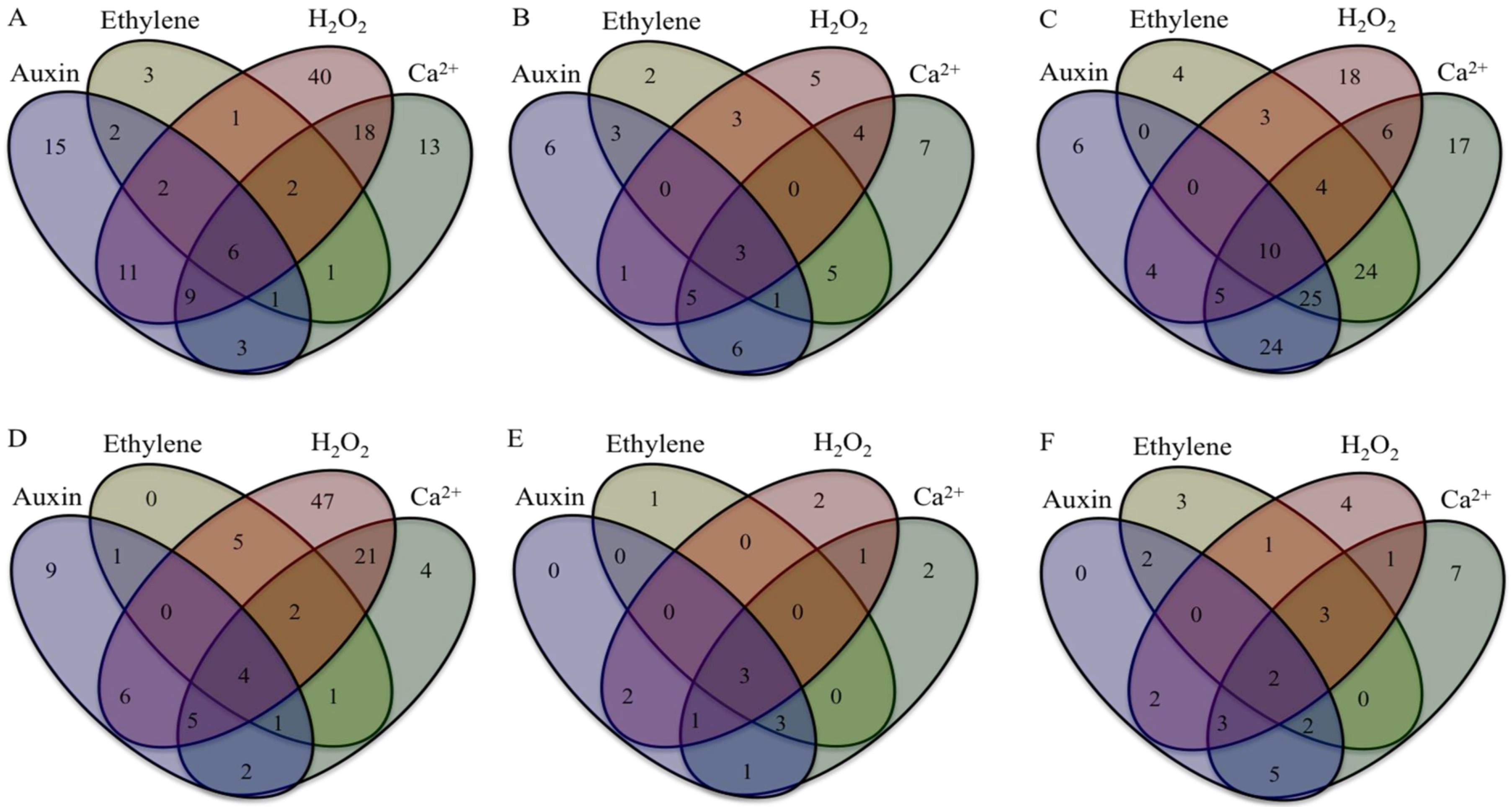

Supplementary Figure S6. Venn diagrams showing numbers of signal-sensitive ETC-specific DEGs involved in signalling that were (A – C) up-regulated or (D – F) down-regulated specifically during (A, D) UWL construction, (B, E) WI papillae construction, (C, F) shared between UWL and WI papillae construction phases. Significant impacts of pharmacological agents on DEG expression levels were determined by comparing mean expression levels (RPKM) of corresponding DEGs from cotyledons cultured in the presence versus absence of each agent using an unpaired, unequal sample size, two tailed t-test ($p \leq 0.05$). Expression data were generated from batches of cotyledons cultured in the absence (n = 6 batches) and presence (n = 3 batches) of the specified pharmacological signal blocker.

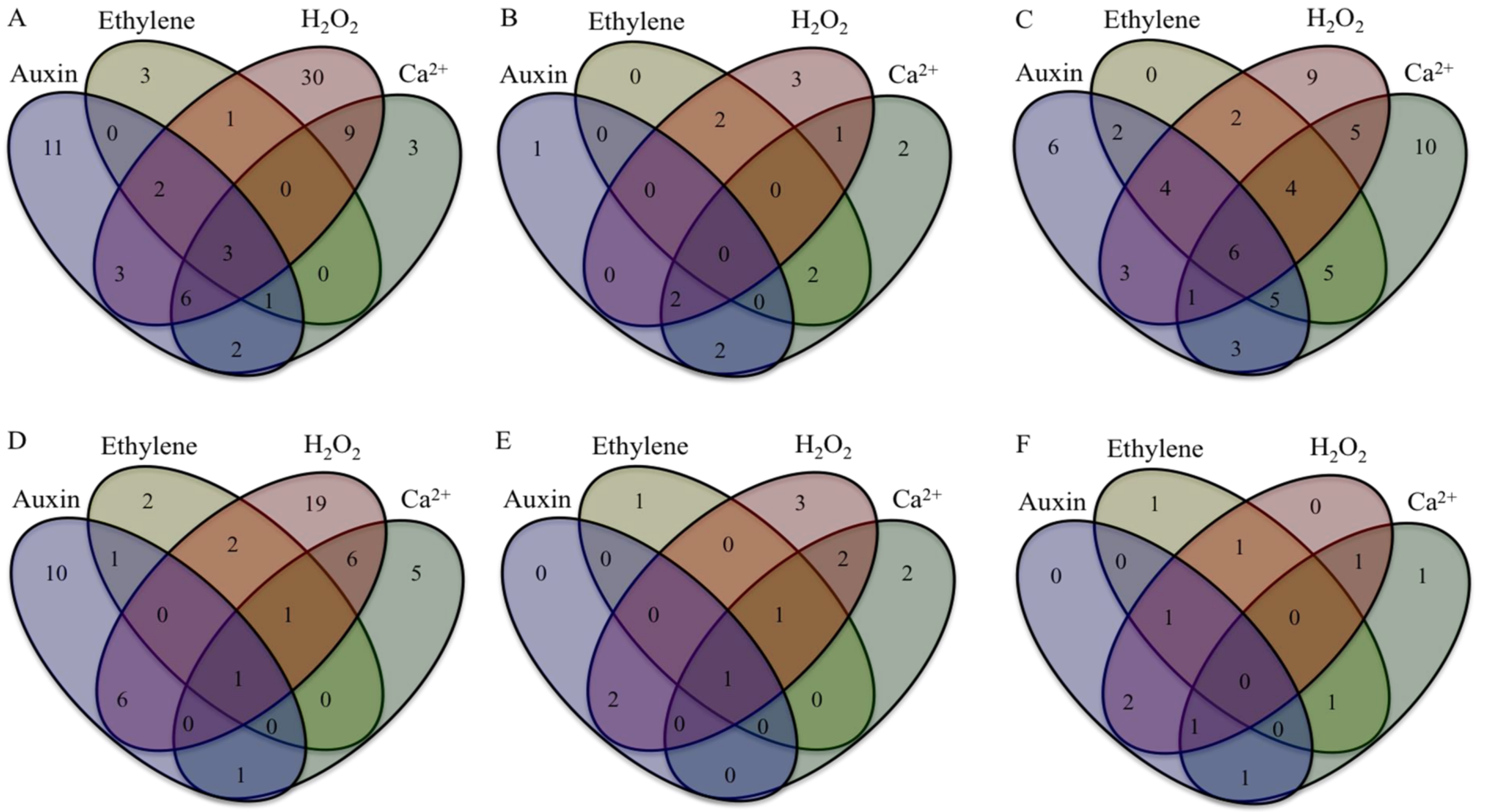

Supplementary Figure S7. Venn diagrams showing numbers of signal-sensitive ETC-specific DEGs involved in intracellular organization that were (A – C) up-regulated or (D – F) down-regulated specifically during (A, D) UWL construction, (B, E) WI papillae construction, (C, F) shared between UWL and WI papillae construction phases. Significant impacts of pharmacological agents on DEG expression levels were determined by comparing mean expression levels (RPKM) of corresponding DEGs from cotyledons cultured in the presence versus absence of each agent using an unpaired, unequal sample size, two tailed t-test ($p \leq 0.05$). Expression data were generated from batches of cotyledons cultured in the absence (n = 6 batches) and presence (n = 3 batches) of the specified pharmacological signal blocker.

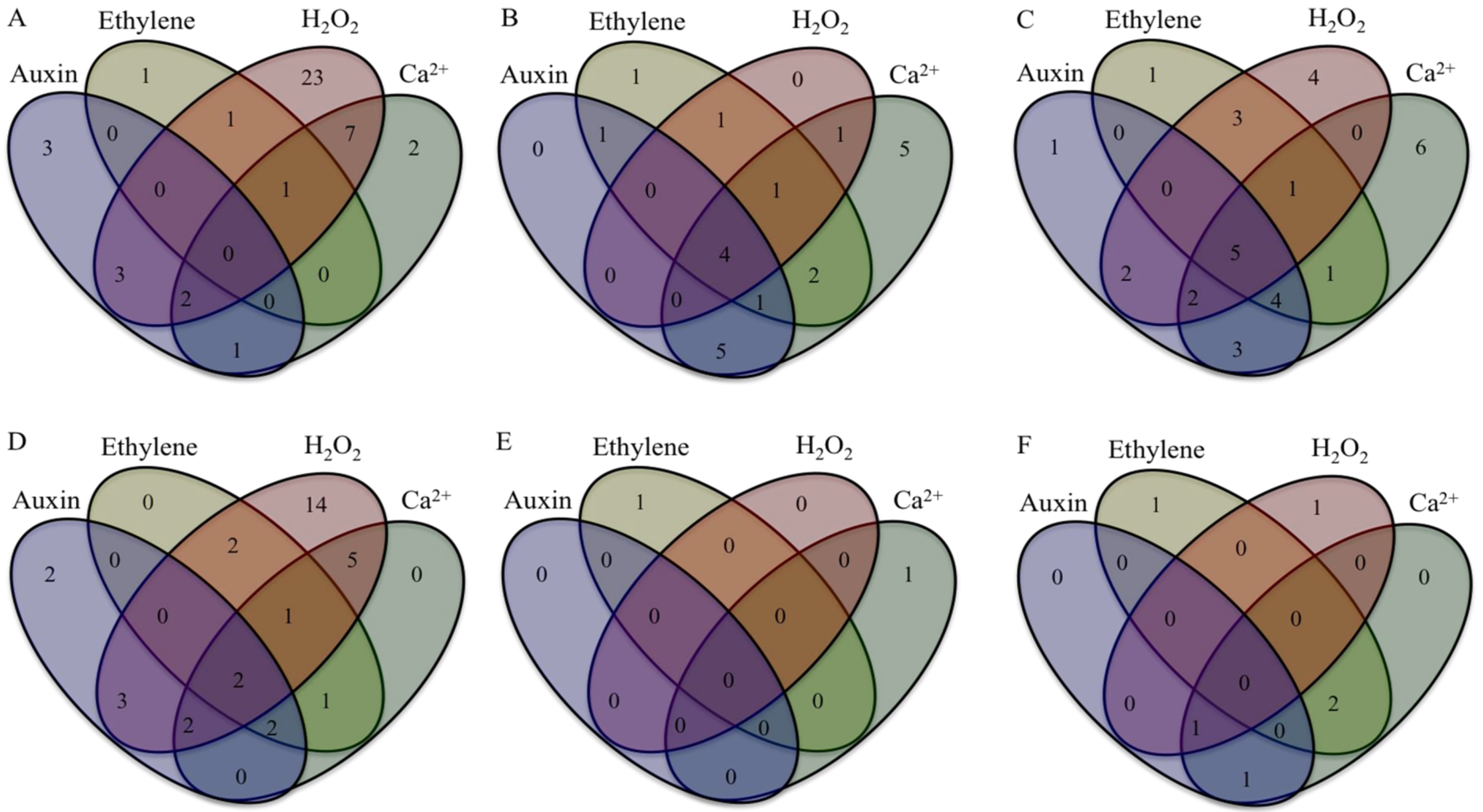

Supplementary Figure S8. Venn diagrams showing numbers of signal-sensitive ETC-specific DEGs involved in cell wall construction that were (A – C) up-regulated or (D – F) down-regulated specifically during (A, D) UWL construction, (B, E) WI papillae construction, (C, F) shared between UWL and WI papillae construction phases. Significant impacts of pharmacological agents on DEG expression levels were determined by comparing mean expression levels (RPKM) of corresponding DEGs from cotyledons cultured in the presence versus absence of each agent using an unpaired, unequal sample size, two tailed t-test ($p \leq 0.05$). Expression data were generated from batches of cotyledons cultured in the absence (n = 6 batches) and presence (n = 3 batches) of the specified pharmacological signal blocker.

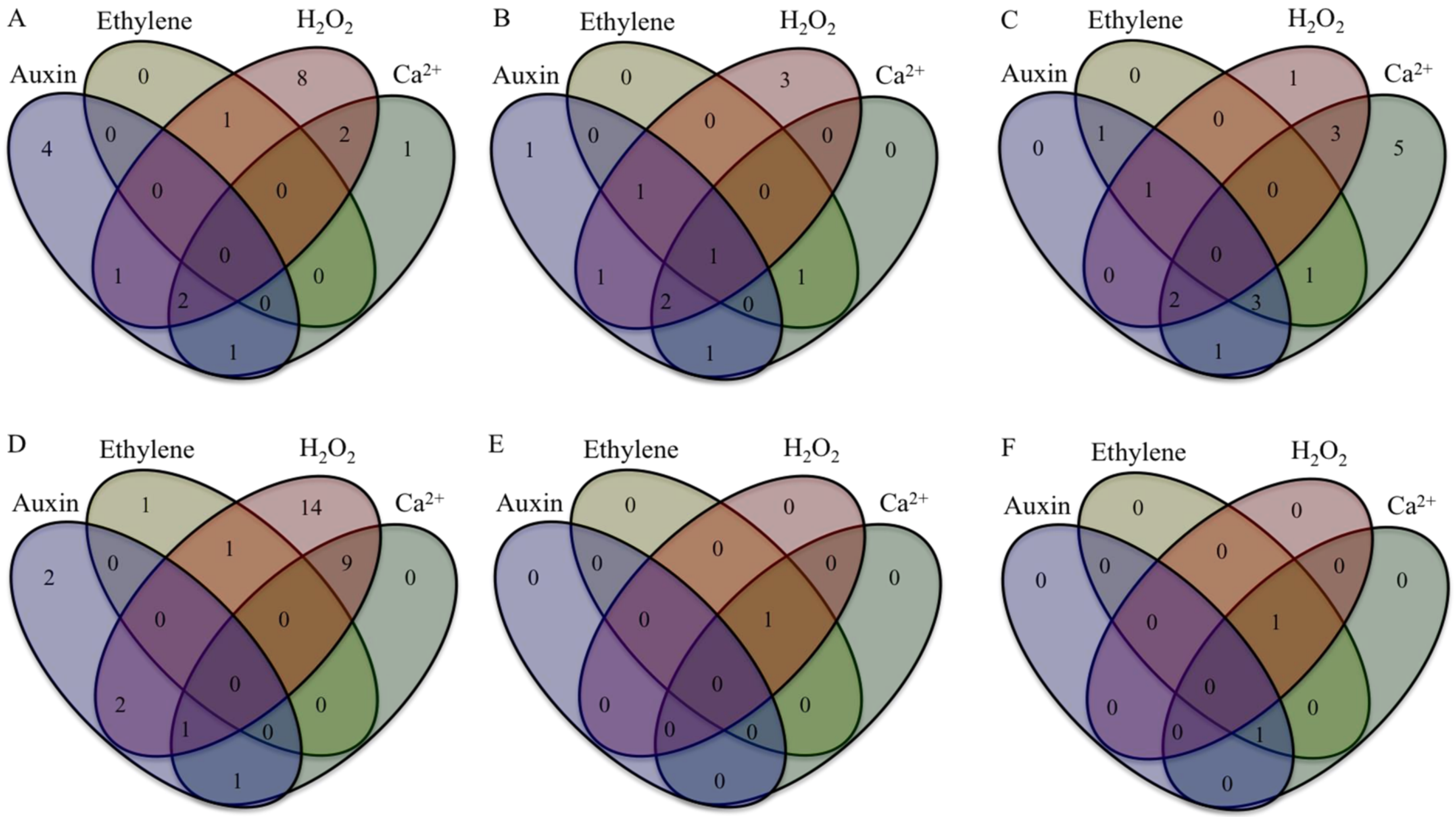

Supplementary Figure S9. Venn diagrams showing numbers of signal-sensitive ETC-specific DEGs involved in transport of nutrients that were (A – C) up-regulated or (D – F) down-regulated specifically during (A, D) UWL construction, (B, E) WI papillae construction, (C, F) shared between UWL and WI papillae construction phases. Significant impacts of pharmacological agents on DEG expression levels were determined by comparing mean expression levels (RPKM) of corresponding DEGs from cotyledons cultured in the presence versus absence of each agent using an unpaired, unequal sample size, two tailed t-test ($p \leq 0.05$). Expression data were generated from batches of cotyledons cultured in the absence (n = 6 batches) and presence (n = 3 batches) of the specified pharmacological signal blocker.
