# Supplementary material for: Transcript Profiling Identifies Gene Cohorts Controlled by Each Signal Regulating Trans-Differentiation of Epidermal Cells of Vicia faba Cotyledons to a Transfer Cell Phenotype
Source: Front Plant Sci. 2017 Nov 28;8:2021. doi: 10.3389/fpls.2017.02021 (PMC5712318; doi:10.3389/fpls.2017.02021)
Supplement: Supplementary file 1 [file Data_Sheet_1.ZIP › Supplementary files FF pdfs only/Supplementary Table S1 .pdf]

Supplementary Table S1. Primer sequences used for RT-qPCR determination of expression levels of specified genes to validate RNA-sequencing data sets.

Gene group	Gene ID	Forward primer	Reverse primer
Housekeeping genes	*VfEF2α*	GACAACATGATTGAGAGGTCCACC	GGCTCCTTCTCAATCTCCTTACC
	VfNADHD4	AGGGTTAGTGAGCACCATGC	ATAGCCAAAGGGAATACGCC
	Vf60SL2	GATTCTCGATGCAGGGCTAC	CCACTGCTCTTGCTCTTTCC
	VfPPlaseG	ACCGAAAGAGGAGCAGAACA	GTACTCCGAACTGGAGAGCG
Uniform wall layer DEGs	*CL6498C2*	AAGATAACTGCCTGCCAAGCT	GTGGAGATCGAAGGAACCGTT
	CL6949C2	TCCTCAAATGGTCAAGAGGGC	GTACAACTACGAGTGCGCCTA
	CL10622C1	TAGCGATTACGGAATGTCGGG	TGGACTTGGATCCTTGGAAGC
	U7484	TCACATGACGCCATAACCATA	TTCCAAGGCTAAGATCAAGCA
	U9449	CTTTCCTTGTCGGATCGCGAA	AGCCTACCAAAGCCCACAAAC
	U12508	TTGCGAACATGGATCAAACTC	CCCTCTTCCACAAAACACACA
	U14740	ACGCAGCTGTAGTTTTCCAGA	GTCATCAGCCAAATCAGGTGC
	U23619	AGGATCAGCCAAACAAAACGC	GGGACTTGCAGAGAATCAGCT
	U24743	CATTCCCGACCAATCTGTGCA	CAGGACATGGCCAAGATTCCC
Wall ingrowth papillae DEGs	*U830*	TGAGTTGATCGGCGTTGAAGA	TCGTGTGCGGACATTCTTACA
	U919	CATCCAAGACAGTGACGGGAA	CCGCACTTCCCACTAAGAGTT
	U23718	CAGACGACAACCCAGTGATCA	CATACTTGTACGCGCTTGCTC
	U26632	ATGTCTCCGGTGGCTTTTGAT	CCCACCAGCACAAACAGAATG
	U29153	CATTAGGTGCTTTGGCTCTTG	TTCTCCAACTTCCTTGCATTG
