# Supplementary material for: Transcript Profiling Identifies Gene Cohorts Controlled by Each Signal Regulating Trans-Differentiation of Epidermal Cells of Vicia faba Cotyledons to a Transfer Cell Phenotype
Source: Front Plant Sci. 2017 Nov 28;8:2021. doi: 10.3389/fpls.2017.02021 (PMC5712318; doi:10.3389/fpls.2017.02021)
Supplement: Supplementary file 1 [file Data_Sheet_1.ZIP › Supplementary files FF pdfs only/Supplementary Table S10.pdf]

Supplementary Table S10. Effect of auxin and ethylene on WI papillae construction in *trans*-differentiating adaxial epidermal cells of cultured *V. faba* cotyledons. Cotyledons were cultured for 9 h to ensure deposition of the uniform wall layer (Zhang *et al.*, 2015d) prior to transferring cotyledons to media with/without exo-/endocytosis inhibitors at 4°C for 4 h and thereafter for a further 6 h of culture at 26°C. Inhibitors tested were the auxin signalling inhibitor, p-chlorophenoxyisobutyric acid (PCIB) and ethylene biosynthesis inhibitor, aminoethoxyvinylglycine (AVG). Statistical significance between the data sets was tested using paired t-test ($p \leq 0.05$). Data are means ± SEs of percent cells with WI papillae determined from 100 cells per cotyledon and six replicate cotyledons (n = 6). Data with different letters are significantly different from the 9 h control mean.

Treatment	% cells with WI papillae
9 h Control	61 ±2 [a]
9 h + 6 h Control	86 ± 3 [b]
9 h + 6 h PCIB (200 μM)	59 ± 3 [a]
9 h + 6 h AVG (100 μM)	76 ± 3 [c]
