# Supplementary material for: Transcript Profiling Identifies Gene Cohorts Controlled by Each Signal Regulating Trans-Differentiation of Epidermal Cells of Vicia faba Cotyledons to a Transfer Cell Phenotype
Source: Front Plant Sci. 2017 Nov 28;8:2021. doi: 10.3389/fpls.2017.02021 (PMC5712318; doi:10.3389/fpls.2017.02021)
Supplement: Supplementary file 1 [file Data_Sheet_1.ZIP › Supplementary files FF pdfs only/Supplementary Table S2 .pdf]

Supplementary Table S2. Pharmacological reagents selected to block signals regulating *trans*-differentiation of *V. faba* cotyledon epidermal cells to a TC phenotype.

Pharmacological agent and media concentration	Signal blocked	Effect	Reference
200 µM p-chlorophenoxyisobutyric acid (PCIB)	Auxin	Inhibits auxin action	Oono et al., (2003); Dibley et al., (2009)
100 µM aminoethoxyvinylglycine (AVG)	Ethylene	Inhibits biosynthesis of ethylene precursor, 1-aminocyclopropane-1-carboxylic acid (ACC)	Konze et al., (1980); Zhou et al., (2010)
10 mM ascorbic acid (AA)	Extracellular hydrogen peroxide (H_2O_2)	Membrane impermeable H_2O_2 scavenger that blocks formation of the polarized extracellular H_2O_2 signal	Foyer and Noctor, (2005); Andriunas et al., (2012); Xia et al., (2012)
600 µM 1, 2-bis(o-aminophenoxy)ethane-N,N,N',N'-tetraacetic acid (BAPTA)	Intracellular calcium ($[Ca^{2+}]_{cyt}$)	Chelates extracellular Ca^{2+} to block generation of an elevated $[Ca^{2+}]_{cyt}$ signal	Oiki et al., (1994); Zhang et al., (2015a)

References:

Andriunas, F.A., Zhang, H.M., Xia, X., Offler, C.E., McCurdy, D.W. and Patrick, J.W. (2012) Reactive oxygen species form part of a regulatory pathway initiating *trans*-differentiation of epidermal transfer cells in *Vicia faba* cotyledons. *J. Exp. Bot.* 63: 3617-3629.

Dibley, S.J., Zhou, Y., Andriunas, F.A., Talbot, M.J., Offler, C.E., Patrick, J.W., "et al.," (2009) Early gene expression programs accompanying trans-differentiation of epidermal cells of *Vicia faba* cotyledons into transfer cells. *New Phytol.* 182: 863-877.

Foyer, C.H., and Noctor, G. (2005) Redox homeostasis and antioxidant signaling: a metabolic interface between stress perception and physiological responses. Plant Cell 17: 1866-1875.

Konze, J.R., Jones, J.F., Boller, T., and Kende, H. (1980) Effect of 1-Aminocyclopropane-1-Carboxylic Acid on the Production of Ethylene in Senescing Flowers of *Ipomoea tricolor Cav. Plant Physiol.* 66: 566-571.

Oiki, S., Yamamoto, T., and Okada, Y. (1994) A simultaneous evaluation method of purity and apparent stability constant of Ca-chelating agents and selectivity coefficient of Ca-selective electrodes. *Cell Calcium* 15: 199-208.

Oono, Y., Ooura, C., Rahman, A., Aspuria, E.T., Hayashi, K., Tanaka, A., "et al.," (2003) p-Chlorophenoxyisobutyric acid impairs auxin response in Arabidopsis root. *Plant Physiol.* 133: 1135-1147.

Xia, X., Zhang, H-M., Andriunas, F.A., Offler, C.E. and Patrick, J.W. (2012) Extracellular hydrogen peroxide, produced through a respiratory burst/superoxide dismutase pathway, directs ingrowth wall formation in epidermal cells of *Vicia faba* cotyledons. *Plant Signal. Behav.* 7: 1125-1128.

Zhang, H-M., Imtiaz, M.S., Laver, D.R., McCurdy, D.W., Offler, C.E., van Helden, D.F., "et al.," (2015a) Polarized and persistent Ca^{2+} plumes define loci for formation of wall ingrowth papillae in transfer cells. *J. Exp. Bot.* 66: 1179-1190.

Zhou, Y., Andriunas, F.A., Offler, C.E., McCurdy, D.W. and Patrick, J.W. (2010) An epidermal-specific ethylene signal cascade regulates *trans*-differentiation of transfer cells in *Vicia faba* cotyledons. *New Phytol.* 185: 931-943.
