# Supplementary material for: Transcript Profiling Identifies Gene Cohorts Controlled by Each Signal Regulating Trans-Differentiation of Epidermal Cells of Vicia faba Cotyledons to a Transfer Cell Phenotype
Source: Front Plant Sci. 2017 Nov 28;8:2021. doi: 10.3389/fpls.2017.02021 (PMC5712318; doi:10.3389/fpls.2017.02021)
Supplement: Supplementary file 1 [file Data_Sheet_1.ZIP › Supplementary files FF pdfs only/Supplementary Table S3 251017FF.pdf]

Supplementary Table S3. Effect of pharmacological inhibition of each signal on percentages of viable epidermal cells and those exhibiting WI papillae. For the current study cotyledons were cultured for 12 h on separate media containing one of the following pharmacological agents: 200 µM PCIB (auxin action blocker); 100 µM AVG (ethylene biosynthesis inhibitor); 10 mM ascorbic acid (extracellular H_2O_2 scavenger); 600 µM BAPTA (extracellular Ca^{2+} chelator). For the presented published findings, unless specified otherwise, cotyledons were cultured for 15 h on media ± pharmacological agent or on media containing the pharmacological agent together with the signal supplied exogenously to test for agent specificity. Means ± SEs of six replicate cotyledons per treatment with 100 cells scored per replicate cotyledon.

**Study design**	**Parameter measured**	**Signal pharmacologically inhibited/recovered:**					**Reference**
		**None (Control)**	**Auxin**	**Ethylene**	**Extracellular H_2O_2**	**Cytosolic Ca^{2+}**	
**Current study**							
± pharmacological agent	% cells with WIs	81.5 ± 2.4	14.6 ± 0.7	14.7 ± 2.3	20.5 ± 3.3	15.7 ± 1.5	
	% viable cells	98.8 ± 0.7	97.8 ± 0.8	98.2 ± 0.8	97.8 ± 0.7	98.9 ± 0.7	
**Published findings + experimental design**							
± PCIB (auxin)	% cells with WIs	94.6 ± 0.9	36.5 ± 5.3				Dibley et al., (2009); Zhou et al., (2010)
PCIB +ACC (ethylene recovery)	% cells with WIs		95.0 ± 1.9				
± AVG (ethylene)	% cells with WIs	84.5 ± 6.3		17.6 ± 1.8			Zhou et al., (2010)
AVG + ACC (ethylene recovery)	% cells with WIs			70.4 ± 3.8			
± AA (H_2O_2)	% cells with WIs	82.7 ± 6.3			17.1 ± 5.7		Andriunas et al., (2012)
± DPI (H_2O_2)	% cells with WIs	71.1 ± 2.7			8.1 ± 2.2		
DPI + H_2O_2 (recovery)	% cells with WIs				61.2 ± 4.4		
± BAPTA (Ca^{2+})	% cells with WIs	86.8 ± 6.7				25.1 ± 1.7	Zhang et al., (2015a)
BAPTA (15 h) + 15 h BAPTA free (recovery)	% cells with WIs					96.9 ± 3.3	

References:

Andriunas, F.A., Zhang, H.M., Xia, X., Offler, C.E., McCurdy, D.W. and Patrick, J.W. (2012) Reactive oxygen species form part of a regulatory pathway initiating *trans*-differentiation of epidermal transfer cells in *Vicia faba* cotyledons. *J. Exp. Bot.* 63: 3617-3629.

Dibley, S.J., Zhou, Y., Andriunas, F.A., Talbot, M.J., Offler, C.E., Patrick, J.W., "et al.," (2009) Early gene expression programs accompanying trans-differentiation of epidermal cells of *Vicia faba* cotyledons into transfer cells. *New Phytol.* 182: 863-877.

Zhang, H-M., Imtiaz, M.S., Laver, D.R., McCurdy, D.W., Offler, C.E., van Helden, D.F., "et al.," (2015a) Polarized and persistent Ca^{2+} plumes define loci for formation of wall ingrowth papillae in transfer cells. *J. Exp. Bot.* 66: 1179-1190.

Zhou, Y., Andriunas, F.A., Offler, C.E., McCurdy, D.W. and Patrick, J.W. (2010) An epidermal-specific ethylene signal cascade regulates *trans*-differentiation of transfer cells in *Vicia faba* cotyledons. *New Phytol.* 185: 931-943.
