# Supplementary material for: Transcript Profiling Identifies Gene Cohorts Controlled by Each Signal Regulating Trans-Differentiation of Epidermal Cells of Vicia faba Cotyledons to a Transfer Cell Phenotype
Source: Front Plant Sci. 2017 Nov 28;8:2021. doi: 10.3389/fpls.2017.02021 (PMC5712318; doi:10.3389/fpls.2017.02021)
Supplement: Supplementary file 1 [file Data_Sheet_1.ZIP › Supplementary files FF pdfs only/Supplementary Table S4 .pdf]

Supplementary Table S4. Number of reads generated from Illumina sequencing used to identify gene cohorts expressed in adaxial epidermal cells of *V. faba* cotyledons. Cotyledons were freshly harvested (T = 0) or cultured for 3 or 12 h on separate media containing one of the following pharmacological treatments: 200 µM PCIB (auxin action blocker); 100 µM AVG (ethylene biosynthesis inhibitor); 10 mM ascorbic acid (extracellular H_2O_2 scavenger); 600 µM BAPTA (extracellular Ca^{2+} chelator). Only uniquely mapped clean reads were used to determine expression levels of gene cohorts in each cotyledon population.

Sample	Clean reads	% Mapped clean reads	% Uniquely mapped clean reads
T = 0 rep 1	68136744	84%	45%
T = 0 rep 2	59194810	84%	42%
T = 0 rep 3	65587918	84%	54%
3 h Control rep 1	70801376	86%	61%
3 h Control rep 2	64544078	85%	61%
3 h Control rep 3	62851458	85%	63%
3 h PCIB rep 1	69494728	86%	58%
3 h PCIB rep 2	66306730	85%	63%
3 h PCIB rep 3	59881344	85%	61%
3 h AVG rep 1	63635352	86%	62%
3 h AVG rep 2	60623468	85%	59%
3 h AVG rep 3	65013712	85%	64%
3 h AA rep 1	63403664	86%	57%
3 h AA rep 2	63594928	84%	55%
3 h AA rep 3	62106548	84%	59%
3 h BAPTA rep 1	61939368	86%	62%
3 h BAPTA rep 2	61680130	85%	62%
3 h BAPTA rep 3	62727614	84%	62%
12 h Control rep 1	60034772	85%	57%
12 h Control rep 2	67063092	84%	55%
12 h Control rep 3	65746790	84%	57%
12 h PCIB rep 1	63999888	86%	61%
12 h PCIB rep 2	67589356	85%	60%
12 h PCIB rep 3	61905438	85%	59%
12 h AVG rep 1	64863494	85%	59%
12 h AVG rep 2	61739124	85%	58%
12 h AVG rep 3	62206644	85%	54%
12 h AA rep 1	64746716	85%	53%
12 h AA rep 2	63293164	85%	58%
12 h AA rep 3	63533480	86%	55%
12 h BAPTA rep 1	63916600	85%	61%
12 h BAPTA rep 2	68306202	85%	58%
12 h BAPTA rep 3	63803662	85%	59%
