# Supplementary material for: Transcript Profiling Identifies Gene Cohorts Controlled by Each Signal Regulating Trans-Differentiation of Epidermal Cells of Vicia faba Cotyledons to a Transfer Cell Phenotype
Source: Front Plant Sci. 2017 Nov 28;8:2021. doi: 10.3389/fpls.2017.02021 (PMC5712318; doi:10.3389/fpls.2017.02021)
Supplement: Supplementary file 1 [file Data_Sheet_1.ZIP › Supplementary files FF pdfs only/Supplementary Table S5 .pdf]

Supplementary Table S5. Comparison of transcript levels of unigenes in adaxial epidermal cells of *V. faba* cotyledons between controls and data published in Zhang et al., (2015d). Unigenes expressed (mean RPKM >1) in the two data sets at each time point were selected and processed using limmaR to assess whether a unigene shared identical expression levels between the two data sets (FDR corrected p $\leq$0.05). More than 99% of unigenes exhibited comparable expression levels between the two data sets. Those that had significantly different expression levels were excluded from further analysis.

Culture time	Number of unigenes with identical RPKM values in the two data sets	Number of unigenes with different RPKM values in the two data sets
0 h	30150	225
3 h	28997	200
12 h	30595	277
