# Supplementary material for: Transcript Profiling Identifies Gene Cohorts Controlled by Each Signal Regulating Trans-Differentiation of Epidermal Cells of Vicia faba Cotyledons to a Transfer Cell Phenotype
Source: Front Plant Sci. 2017 Nov 28;8:2021. doi: 10.3389/fpls.2017.02021 (PMC5712318; doi:10.3389/fpls.2017.02021)
Supplement: Supplementary file 1 [file Data_Sheet_1.ZIP › Supplementary files FF pdfs only/Supplementary Table S6.pdf]

Supplementary Table S6. Number of differentially expressed genes (DEGs) in adaxial epidermal transfer cells (ETC) and storage parenchyma cells (SPC) of *V. faba* cotyledons across two phases of wall labyrinth construction. Cotyledons were freshly harvested (0 h) or cultured on MS medium for 3 or 12 h and thereafter processed for RNA-seq analysis and DEG identification in three broad temporal expression profiles (see Supplementary Figure S1 and note temporal patterns marked with * and ** were omitted). DEGs were determined using limmaR from six replicate batches of cotyledons for adaxial epidermal cells and edgeR from three replicate batches of cotyledons for storage parenchyma cells.

**Temporal expression pattern coincides with:**	**Pattern of DEGs**	**Total number of DEGs that are:**		
		**ETC specific**	**SPC specific**	**ETC/SPC shared**
UWL deposition (0 to 3 h only)	Up-regulated	3208	1157	601
	Down-regulated	3296	247	98
WI papillae deposition (3 to 12 h only)	Up-regulated	733	746	101
	Down-regulated	307	395	7
Shared UWL and WI (both 0 to 3h and 3 to 12 h)	Up-regulated	1611	218	90
	Down-regulated	587	73	29
