# Supplementary material for: Transcript Profiling Identifies Gene Cohorts Controlled by Each Signal Regulating Trans-Differentiation of Epidermal Cells of Vicia faba Cotyledons to a Transfer Cell Phenotype
Source: Front Plant Sci. 2017 Nov 28;8:2021. doi: 10.3389/fpls.2017.02021 (PMC5712318; doi:10.3389/fpls.2017.02021)
Supplement: Supplementary file 1 [file Data_Sheet_1.ZIP › Supplementary files FF pdfs only/Supplementary Table S7 .pdf]

Supplementary Table S7. List of Mapman and KEGG annotated gene cohorts identified as candidates encoding proteins contributing to TC *trans*-differentiation (signalling, intracellular organization and cell wall) and nutrient transporters.

(A) **Signalling cohort**:

**Mapman/KEGG Pathway Number**	**Pathway Name**
Mapman:	
17	Hormone metabolism
17.1	Hormone metabolism.abscisic acid
17.2	Hormone metabolism.auxin
34.23.1	Transport.hormones.auxin
17.3	Hormone metabolism.brassinosteroid
17.4	Hormone metabolism.cytokinin
17.5	Hormone metabolism.ethylene
17.6	Hormone metabolism.gibberellin
17.7	Hormone metabolism.jasmonate
17.8	Hormone metabolism.salicylic acid
20.1.1	Stress.biotic.respiratory burst
20.2.4	Stress.abiotic.touch/wounding
21.6	Redox.dismutases and catalases
26.12	Misc.peroxidases
30.6	Signalling.MAP kinases
30.3	Signalling.calcium
34.21	Transport.calcium
34.22	Transport.cyclic nucleotide or calcium regulated channels
34.16	Transport.ABC transporters and multidrug resistance systems (only those known to transport auxin)
KEGG:	
4	Plant hormone signal transduction
49	Cysteine and methionine metabolism (only those related to ethylene A biosynthesis)
55	Carotenoid biosynthesis (only those related to ABA biosynthesis)
81	Tryptophan metabolism (only those related to auxin biosynthesis)
88	Diterpenoid biosynthesis (only those related to gibberellin biosynthesis)
72	alpha-Linolenic acid metabolism (only those related to jasmonic acid biosynthesis)
107	Brassinosteroid biosynthesis

(B) **Intracellular organization cohort**:

Mapman/KEGG Pathway Number	Pathway Name
Mapman:	
11.1.8	Lipid metabolism.FA synthesis and FA elongation.acyl CoA ligase
11.1.10	Lipid metabolism.FA synthesis and FA elongation.beta ketoacyl CoA synthase
11.6	Lipid metabolism.lipid transfer proteins etc
26.9	Misc.glutathione S transferases
26.17	Misc.dynamin
26.28	Misc.GDSL-motif lipase
31.1	Cell.organisation
31.1.1.1.1	Cell.organisation.cytoskeleton.actin.actin
31.1.1.1.2	Cell.organisation.cytoskeleton.actin.actin depolymerizing factors
31.1.1.1.3	Cell.organisation.cytoskeleton.actin.actin binding
31.1.1.2	Cell.organisation.cytoskeleton.microtubule
31.1.1.2.5	Cell.organisation.cytoskeleton.microtubule.MAP70
31.1.1.2.6	Cell.organisation.cytoskeleton.microtubule.MAP65
31.1.1.3	Cell.organisation.cytoskeleton.myosin
31.1.1.3.11	Cell.organisation.cytoskeleton.myosin.class XI
31.1.1.3.8	Cell.organisation.cytoskeleton.myosin.class VII
35.1.20	Not assigned.no ontology.formin homology 2 domain-containing protein
31.4	Cell.vesicle transport
11.8.1	Lipid metabolism.exotics (steroids, squalene etc).sphingolipids
KEGG:	
10	Endocytosis
74	SNARE interactions in vesicular transport
85	Steroid biosynthesis
86	Sphingolipid metabolism

(C) **Cell wall cohort:**

Mapman/KEGG Pathway Number	Pathway Name
Mapman:	
3.6	Minor CHO metabolism.callose
3.8.2	Minor CHO metabolism.galactose.alpha-galactosidases
10	Cell wall
10.1	Cell wall.precursor synthesis
10.2	Cell wall.cellulose synthesis
10.3	Cell wall.hemicellulose synthesis
10.3.1	Cell wall.hemicellulose synthesis.xyloglucan
10.3.2	Cell wall.hemicellulose synthesis.glucuronoxylan
10.4	Cell wall.pectin synthesis
10.5	Cell wall.cell wall proteins
10.5.1	Cell wall.cell wall proteins.AGPs
10.5.2	Cell wall.cell wall proteins.proline rich proteins
10.5.3	Cell wall.cell wall proteins.LRR
10.5.4	Cell wall.cell wall proteins.HRGP
10.6	Cell wall.degradation
10.5.5	Cell wall.cell wall proteins.RGP
10.6.2	Cell wall.degradation.mannan-xylose-arabinose-fucose
10.6.1	Cell wall.degradation.cellulases and beta -1,4-glucanases
10.6.3	Cell wall.degradation.pectate lyases and polygalacturonases
10.7	Cell wall.modification
10.8	Cell wall.pectin*esterases
10.8.1	Cell wall.pectin*esterases.PME
10.8.2	Cell wall.pectin*esterases.acetyl esterase
26.3.2	Misc.gluco-, galacto- and mannosidases.beta-galactosidase
26.4.1	Misc.beta 1,3 glucan hydrolases.glucan endo-1,3-beta-glucosidase
26.18	Misc.invertase/pectin methylesterase inhibitor family protein

(D) **Nutrient transporter cohort**:

Mapman/KEGG Pathway Number	Pathway Name
Mapman:	
34.2	Transport.sugars
34.3	Transport.amino acids
34.5	Transport.ammonium
34.12	Transport.metal
34.14	Transport.unspecified cations
34.15	Transport.potassium only those involved in Ca^{2+} transport
34.18	Transport.unspecified anions
