# Supplementary material for: Transcript Profiling Identifies Gene Cohorts Controlled by Each Signal Regulating Trans-Differentiation of Epidermal Cells of Vicia faba Cotyledons to a Transfer Cell Phenotype
Source: Front Plant Sci. 2017 Nov 28;8:2021. doi: 10.3389/fpls.2017.02021 (PMC5712318; doi:10.3389/fpls.2017.02021)
Supplement: Supplementary file 1 [file Data_Sheet_1.ZIP › Supplementary files FF pdfs only/Supplementary Table S8 .pdf]

Supplementary Table S8. Numbers of genes expressed in epidermal transfer cells (ETC) and storage parenchyma cells (SPC) of *V. faba* cotyledons during the two phases of TC *trans*-differentiation. Cotyledons were either freshly harvested (0 h) or cultured in MS medium for 3 or 12 h before processing for RNA-seq. Abundance of transcripts was normalized into RPKM values and only transcripts with a mean RPKM value >1 across six replicate batches of cotyledons was considered as expressed.

**Cell type**	**Number of transcripts with RPKM >1 at:**			
	**0 h**	**3 h**	**12 h**	**Total**
ETC	26911	26379	27642	33423
SPC	19456	23492	23868	27437
