# Supplementary material for: Transcript Profiling Identifies Gene Cohorts Controlled by Each Signal Regulating Trans-Differentiation of Epidermal Cells of Vicia faba Cotyledons to a Transfer Cell Phenotype
Source: Front Plant Sci. 2017 Nov 28;8:2021. doi: 10.3389/fpls.2017.02021 (PMC5712318; doi:10.3389/fpls.2017.02021)
Supplement: Supplementary file 1 [file Data_Sheet_1.ZIP › Supplementary files FF pdfs only/Supplementary Table S9 .pdf]

Supplementary Table S9. Numbers of ETC-specific differentially expressed genes (DEGs) detected across the two phases of wall labyrinth construction not included in Supplementary Figures S6-9. DEGs are classified according to their annotation in Mapman Mercator. DEGs were determined using limmaR from six replicate batches of cotyledons for adaxial epidermal cells and edgeR from three replicate batches of cotyledons for storage parenchyma cells. Percentage proportion of DEGs in each Mapman bin category is presented in parentheses.

Mapman bin category	0 to 3 h specific		3 to 12 h specific		Shared 0 to 3h & 3 to 12 h		Total
	Up-regulated	Down-regulated	Up-regulated	Down-regulated	Up-regulated	Down-regulated	
1:PS	7	49	3	0	3	1	63 (2%)
2:major CHO metabolism	6	23	1	1	3	3	37 (1%)
3:minor CHO metabolism	8	17	5	2	7	3	42 (1%)
4:glycolysis	4	18	1	0	4	3	30 (1%)
5:fermentation	3	2	0	0	0	0	5 (0%)
6:gluconeogenesis / glyoxylate cycle	0	5	1	0	0	0	6 (0%)
7:OPP	2	2	1	0	2	0	7 (0%)
8:TCA / org transformation	11	7	3	2	3	0	26 (1%)
9:mitochondrial electron transport / ATP synthesis	6	6	0	0	3	0	15 (1%)
10:cell wall	0	0	0	0	0	0	0 (0%)
11:lipid metabolism	40	20	14	3	29	3	109 (3%)
12:N-metabolism	0	3	0	0	1	3	7 (0%)
13:amino acid metabolism	14	40	7	2	14	1	78 (2%)
14:S-assimilation	2	2	0	0	2	1	7 (0%)
15:metal handling	2	11	1	1	0	4	19 (1%)
16:secondary metabolism	22	28	25	2	58	16	151 (5%)
17:hormone metabolism	0	0	0	0	0	0	0 (0%)
18:Co-factor and vitamin metabolism	8	4	2	0	2	1	17 (1%)
19:tetrapyrrole synthesis	0	10	0	0	3	0	13 (0%)
20:stress	128	41	25	8	61	16	289 (9%)
21:redox	22	15	5	1	11	2	56 (2%)
22:polyamine metabolism	0	1	1	2	0	0	4 (0%)
23:nucleotide metabolism	14	17	1	0	6	1	39 (1%)
24:biodegradation of Xenobiotics	2	3	1	0	0	0	6 (0%)

25:C1-metabolism	0	3	0	0	1	1	5 (0%)
26:misc	59	52	20	6	66	14	217 (7%)
27:RNA	152	209	44	23	105	30	563 (18%)
28:DNA	18	60	11	2	14	3	108 (3%)
29:protein	254	158	53	13	111	22	611 (19%)
30:signalling	85	88	13	5	71	8	270 (9%)
31:cell	7	19	2	0	6	1	35 (1%)
32:micro RNA, natural antisense etc	0	0	0	0	0	0	0 (0%)
33:development	32	48	9	6	26	21	142 (5%)
34:transport	66	46	17	7	49	18	203 (6%)
Total	974	1007	266	86	661	176	3170 (100%)
